# Initial spliceosomal U4/U6 di-snRNA formation occurs in the cytoplasm of *Saccharomyces cerevisiae* and requires a guard protein mediated quality control

**DOI:** 10.1093/nar/gkaf1500

**Published:** 2026-01-08

**Authors:** Xiaoxiao Wang, Jian Guo, Jing Li, Heike Krebber

**Affiliations:** Abteilung für Molekulare Genetik, Institut für Mikrobiologie und Genetik, Göttinger Zentrum für Molekulare Biowissenschaften (GZMB), Georg-August Universität Göttingen, 37077 Göttingen, Germany; Abteilung für Molekulare Genetik, Institut für Mikrobiologie und Genetik, Göttinger Zentrum für Molekulare Biowissenschaften (GZMB), Georg-August Universität Göttingen, 37077 Göttingen, Germany; Abteilung für Molekulare Genetik, Institut für Mikrobiologie und Genetik, Göttinger Zentrum für Molekulare Biowissenschaften (GZMB), Georg-August Universität Göttingen, 37077 Göttingen, Germany; Abteilung für Molekulare Genetik, Institut für Mikrobiologie und Genetik, Göttinger Zentrum für Molekulare Biowissenschaften (GZMB), Georg-August Universität Göttingen, 37077 Göttingen, Germany

## Abstract

Unlike mRNA surveillance, ncRNA quality control is less well understood. While mRNA maturation is monitored by guard proteins that allow nuclear export of correctly processed transcripts or retention and degradation of faulty RNAs, such surveillance system is unknown for ncRNAs. This study investigates the maturation process of the snRNA U6 in *Saccharomyces cerevisiae*, revealing that this RNAPIII transcript undergoes quality control by established guard proteins and the novel factor Lhp1 (human La), which ensures proper loading of the Lsm-ring in the nucleus. Subsequent Mex67 binding facilitates the nuclear export of pre-U6. In the cytoplasm, pre-U6 associates with Prp24 which assists in annealing with pre-U4. Defects in di-snRNP formation are identified by the guard proteins Npl3, Gbp2, and Hrb1. These proteins retain the RNA in the cytoplasm and recruit Dcp1 and Dcp2 for de-capping, along with Xrn1 for degradation of faulty pre-U6. Correctly assembled U4/U6 complexes are released from the guard proteins and imported back into the nucleus. This guard protein-mediated surveillance mechanism prevents faulty di-snRNPs to torpedo the spliceosome, underscoring the significance of the compartmented maturation and quality control of ncRNA. Additionally, the study illustrates that RNA surveillance mechanisms extend beyond coding RNAs and involve similar quality control mechanisms and proteins.

## Graphical Abstract

**Graphical Abstract:**
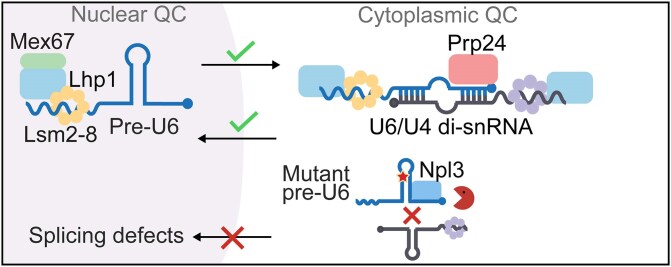

## Introduction

Spliceosome assembly occurs on intron containing pre-mRNAs in the nucleus [1–3]. Several proteins as well as five small nuclear ribonucleoparticles (snRNPs) are loaded and act in a stepwise process to activate the spliceosome. Each snRNP contains one snRNA, one of which, U6 (*U6*/*SNR6*), is generated by RNA-polymerase III (RNAPIII) in yeast and four of which, U1, U2, U4, and U5 (*U1*/*SNR19, U2*/*LSR1, U4*/*SNR14*, and *U5/SNR7*) are RNAPII transcripts [4, 5]. These RNAPII transcripts are, like pre-mRNAs, protected from nuclease attacks by an m^7^G cap at their 5′ ends [3]. The cap in turn is bound by the cap-binding complex (CBC), which interacts with the export receptor Xpo1/Crm1 [6]. Nuclear export is further supported by the export receptor heterodimer Mex67–Mtr2 (short Mex67) that is recruited upon RNA surveillance by the guard proteins [6, 7]. The more guard proteins have recruited Mex67, the more likely is the nuclear export of the RNA [8, 9]. Guard proteins were very well studied for mRNAs and it was shown that Npl3 surveils the capping reaction, Gbp2 and Hrb1 monitors splicing, Hrp1 controls the 3′ cleavage reaction and Nab2 the attachment of the poly(A) tail [10–14]. Upon proper quality control, Mex67 binds to the guard proteins and the RNA can leave the nucleus through the hydrophobic interior of the nuclear pore complex (NPC). Displacement of Mex67 occurs at the cytoplasmic site of the NPC, where the RNA helicase Dbp5 is located, to establish directionality of the transport event [15]. On messenger RNAs (mRNAs), the guard proteins subsequently leave the RNA in initial rounds of translation [16]. Export of the snRNAs was also shown to depend on Xpo1 and Mex67 that contacts the CBC on m^7^G-caps [6]. Furthermore, binding of the guard proteins Npl3, Gbp2, and Hrb1 to all snRNAs was shown before [6]; however, their particular functions in snRNA quality control are currently unclear. As RNAPII produced snRNAs are capped, it is likely that Npl3 surveils 5′ capping, but the role for Gbp2 and Hrb1 are unknown. Upon arrival in the cytoplasm, the RNAPII generated pre-snRNAs receive an Sm-ring, which is necessary to restrict the sequential formation of snRNP cores, the upcoming 3′ RNA processing and to generate mature snRNAs [6, 17]. This Sm-ring attachment serves as a quality control checkpoint, as it allows the binding of the nuclear import receptors Cse1 and Mtr10 [6]. This is very similar to the nuclear re-import of the non-coding RNA (ncRNA) *TLC1* of the telomerase. Physical interaction of the Sm-ring and the import receptors allow to shuttle this ncRNA back into the nucleus [18]. Upon nuclear arrival of the pre-snRNAs, 3′ processing requires an initial Rnt1-mediated cleavage of a stem-loop structure [19]. Subsequently, further 3′ trimming by the Rrp6-containing nuclear exosome occurs up to the Sm-ring, which protects from full degradation [6, 20, 21]. The snRNAs are finally transported into the nucleolus, in which the m^7^G cap is trimethylated by Tgs1. This TMG cap prevents the re-association of the export receptor Xpo1/Crm1 for recurrence of nuclear export [6]. Importantly, preventing nuclear export allows interaction of immature snRNAs with the spliceosome causing mis-splicing [6]. Thus, the nucleo-cytoplasmic shuttling pathway of the snRNAs ensures correct and fully active spliceosomes.

The described nucleo-cytoplasmic shuttling pathway was shown to be used by the RNAPII-generated snRNAs U1, U2, U4, and U5. However, U6 is generated by RNAPIII in yeast and such transcripts, to which also pre-tRNAs belong, receive a short oligo(U) tail. The tail contains a *cis*-diol (a 2′ and 3′ hydroxyl group) at the 3′ end, which is bound by Lhp1 and an Lsm-ring [19, 22–24]. Lhp1 preferentially binds a *cis*-diol rather than a 3′ phosphate, whereas the Lsm-ring, composed of Lsm2-8 binds both a *cis*-diol and a 3′ phosphate. However, as it preferentially binds a 3′ phosphate it was suggested that the exchange of the *cis*-diol to a 3′ phosphate might remove the binding of Lhp1 and increases the binding affinity of the Lsm-ring to U6 [25]. It is currently unknown why Lhp1 has to bind first. Contrary to the known m^7^G-cap of RNAPII generated snRNAs, the structure of the U6 5′ cap in yeast is currently unclear. Importantly, U6, like the other snRNAs was shown to shuttle into the cytoplasm, as it is retained in mutants of *MEX67* and *XPO1* and to bind the export receptor recruiting guard proteins Npl3, Gbp2, and Hrb1 [6]. Since U6 contains no apparent feature that was already shown to be controlled by the guard proteins as it has no m^7^G cap, it is unclear how U6 depends on Mex67 for its nuclear export. Consistent with lacking an m^7^G cap, Xpo1 was shown not to be responsible for its nuclear export [6]. Interestingly, the nuclear re-import of U6 is also dependent on the import receptors Mtr10 and Cse1 [6]; however, it does not receive an Sm-ring. It seems possible that also the Lsm-ring might be able to interact with the import receptors. However, this remains to be shown.

Interestingly, unprocessed pre-U6, which is trimmed by the exoribonuclease Usb1 to become mature U6, accumulates in the cytoplasm of the Sm-ring mutant *smb smd1* at its nonpermissive temperature [6]. As this mutation prevents the Sm-ring formation, which is not directly relevant for U6, this finding might hind toward the possibility that the U4/U6 di-snRNP formation might occur in the cytoplasm. Thus, pre-U6 might be re-imported together with unprocessed pre-U4 and both might undergo full trimming only after re-import. In fact, U4 processing occurs via the nuclear endoribonuclease Rnt1 and the exoribonuclease Rrp6. Nonetheless, this still needs to be demonstrated.

U4/U6 di-snRNP formation requires Prp24, which localizes to the nucleus [26, 27]. Therefore, current literature assumes that the di-snRNP formation takes place in the nucleus [28–30]. For the annealing of U4 and U6, Prp24 binds to the ACAGA-containing sequence motif in U6 (nucleotides 40–58). Thereby, the N-terminal RNA recognition motif (RRM1) of Prp24 is involved in the formation of a positively charged groove, which enhances the binding and annealing of U4 with U6 [26]. Notably, Lhp1 competes with Prp24 for newly synthesized U6 *in vitro* [25], but Prp24 and Lsm2-8 bind cooperatively through a structural domain containing the peptide sequence SNFFL, suggesting that Lhp1 may be exchanged by Prp24. Whether this exchange occurs in the nucleus or in the cytoplasm is currently unclear. Further maturation of pre-U6 requires 3′ to 5′ cleavage by Usb1, which has a dual exoribonuclease and cyclic phosphodiesterase activity that trims back the oligo(U) tail and leaves it with a 3′ phosphate group to avoid over-trimming [25].

In this study, we analyzed the stepwise maturation of U6 and found that this snRNA is exported into the cytoplasm immediately after transcription, in association with the mRNA guard proteins Npl3, Gbp2, and Hrb1, which recruit Mex67 for export. Export is further supported by Lhp1, which also interacts with Mex67 upon pre-U6 binding and surveils as a novel guard protein the correct attachment of the Lsm-ring. In the cytoplasm Prp24 binds pre-U6, which in turn enables pre-U4 to anneal with pre-U6. The di-snRNP is subsequently re-imported back into the nucleus via binding of the pre-U4-attached Sm-ring and the pre-U6-attached Lsm-ring. Both rings contact Mtr10 and Cse1 for re-import through the NPC. Finally, processing of both pre-UsnRNAs occurs and the mature U4/U6 di-snRNP can function in mRNA splicing. In case the di-snRNP formation in the cytoplasm fails, we found that the guard proteins Npl3, Gbp2, and Hrb1 recruit the de-capping factors Dcp1 and Dcp2 and subsequently the 5′-3′ exoribonuclease Xrn1 to U6 for cytoplasmic degradation of the faulty snRNAs, revealing an elaborate compartmental quality control system that ensures proper splicing.

## Results

### Lhp1 is involved in U6 nuclear export by recruiting Mex67

**Figure 1.**
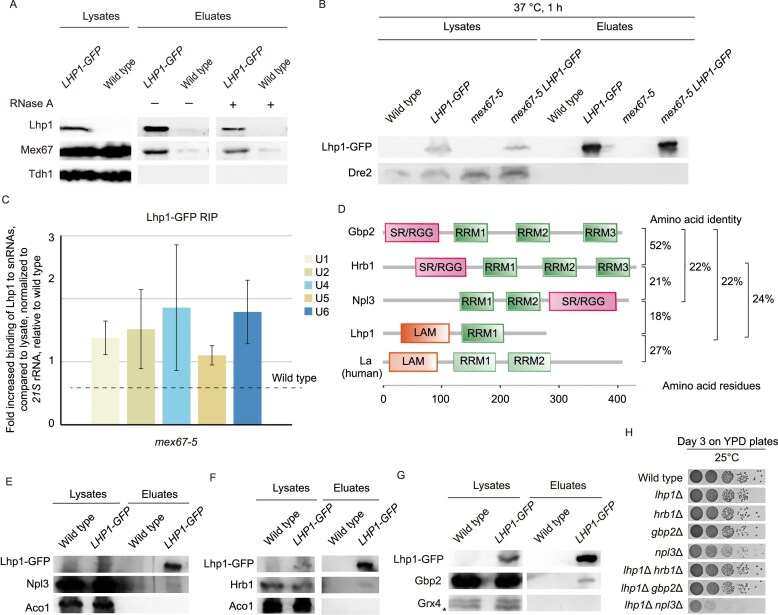
Lhp1 shows similarities to mRNA guard proteins. (**A**) Mex67 and Lhp1 show a physical interaction. Western blot of a co-IP of Mex67 with Lhp1–GFP is shown. The mitochondrial protein Tdh1 served as a washing control; *n* = 3. (**B** and **C**) Lhp1 binding to snRNAs is increased when the nuclear export is blocked. (B) Pulldown of GFP-tagged proteins was validated via western blot analysis. The mitochondrial protein Dre2 served as a negative control for unspecific binding. (C) RNA-co-IP (RIP) experiment with Lhp1 and the result of a subsequent qPCR of the snRNAs is shown from *mex67-5* cells shifted to 37°C for 1 h in comparison to wild-type is shown; *n* = 3. (**D**) Comparison of the domain structures of the yeast guard proteins Gbp2, Hrb1, Npl3, Lhp1, and a homolog of Lhp1 from human cells (La) is shown. (**E–G**) Lhp1 interacts with the guard proteins. (E) Western blot of a co-IP of Npl3 (E), Hrb1 (F), or Gbp2 (G) with GFP-tagged Lhp1 is shown. The mitochondrial proteins Grx4 or Aco1 served as washing controls for unspecific binding; *n* = 3. * indicates a degradation product of Grx4. (**H**) The double knock out of *LHP1* and *NPL3* has a growth defect. The indicated strains were spotted in 10-fold serial dilution onto YPD-full medium plates and were incubated for 3 days at 25°C; *n* = 3.

The mRNA guard proteins Npl3, Gbp2, and Hrb1 were shown to interact not only with mRNAs but also with all five snRNAs, including U6 [6]. Generally, guard proteins recruit the export receptor Mex67 to the mRNAs upon maturation, allowing them to exit the nucleus. The RNAPII transcripts pre-U1, -U2, -U4, and -U5 require also Mex67 for their transport into the cytoplasm, which occurs in support of Xpo1/Crm1, contacting the CBC bound m^7^G cap. In contrast, the RNAPIII transcript pre-U6 does not seem to be affected by Xpo1 [6], suggesting that it does not contain an m^7^G cap. To investigate whether instead other proteins might support the export of pre-U6, we carried out interaction studies of Mex67 with the U6 associated proteins Prp24 and Lhp1. While Prp24 did not show an interaction, a physical contact was detected between Mex67 and Lhp1 (Fig. 1A and Supplementary Fig. S1A and Source Data 1 to Fig. 1). This contact might be either direct or indirect via other proteins. However, it seems not dependent on the presence of RNA. If Lhp1 would be part of the exported pre-U6, one would expect that its binding to this snRNA would not decrease in an export mutant of *MEX67*. Therefore, we shifted the temperature sensitive *mex67-5* mutant for 1 h to 37°C before we analyzed the binding of Lhp1 to U6 in RNA co-immunoprecipitation (RIP) analysis and found a slightly increased interaction, compared to wild-type (Fig. 1B and C and Source Data 1 to Fig. 1). Interaction of Lhp1 not only with U6 but also the other snRNAs was shown earlier [31, 32], and we have demonstrated that also their interactions with Lhp1 do not decrease in *mex67-5* mutants.

Interestingly, Lhp1 is highly homologous to the guard proteins. Remarkably, its amino acid sequence is 24% identical to that of Hrb1 and 22% identical to that of Gbp2, respectively (Fig. 1D and Supplementary Fig. S1C). Besides their ability to recruit Mex67 to RNAs, the guard proteins also interact with each other when bound to RNA [33] (Supplementary Fig. S1B). To investigated whether Lhp1 also interacts with a guard protein, we carried out co-immunoprecipitations (co-IPs) and found slight interactions with Npl3, Hrb1, and Gbp2 (Fig. 1E–G, Supplementary Fig. S1D and Source data 1 to Fig. 1). Besides these physical interactions, we also found that the combination of *npl3∆* with *lhp1*∆ had severe growth defects (Fig. 1H), similar to the genetic interactions detected in the double and triple knock out of *npl3∆, gbp2∆*, and *hrb1∆* [13, 14], which further supports their functional interconnection. Together, these findings suggest that Lhp1 supports the export of correctly assembled pre-U6 through recruitment of Mex67, possibly after jointing nuclear quality control of the ncRNA U6 together with the guard proteins Npl3, Gbp2, and Hrb1.

### Lhp1 controls the assembly of the Lsm-ring in the nucleus

**Figure 2.**
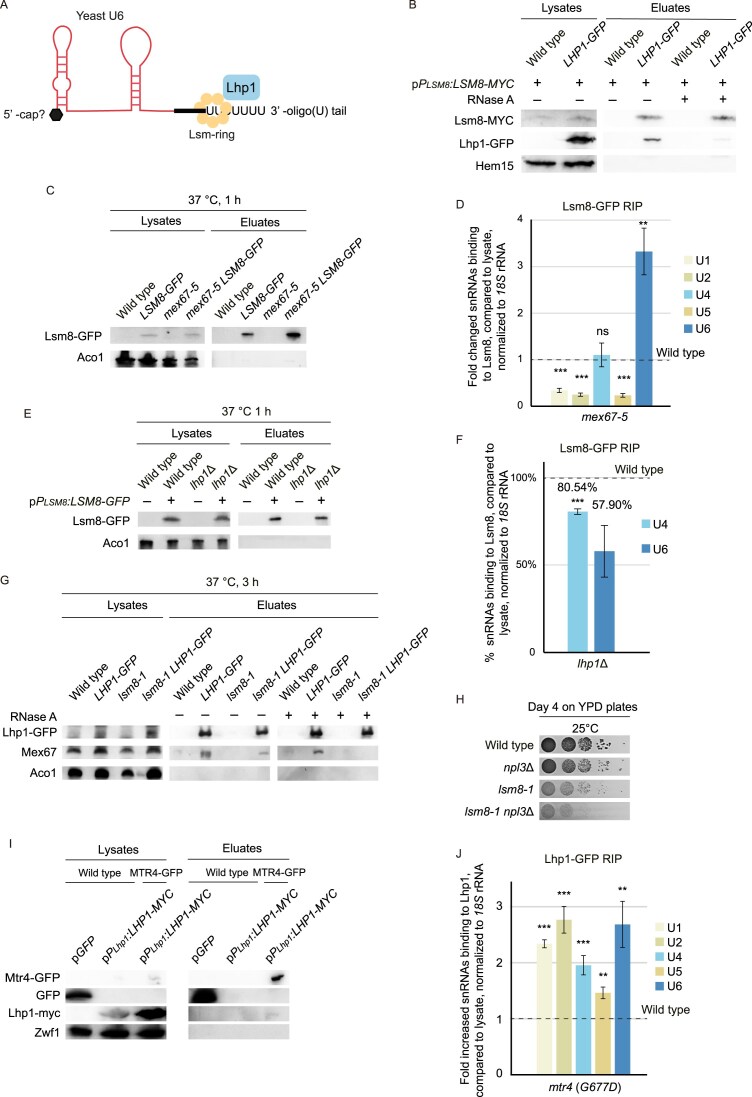
Lsm-ring loading onto U6 in the nucleus is monitored by Lhp1. (**A**) Scheme of the binding site for Lhp1 and the Lsm-ring at the 3′ end of pre-U6. (**B**) Western blot analysis of co-precipitated Lsm8-myc with Lhp1–GFP is shown. The mitochondrial protein Hem15 served as a negative control for unspecific binding; *n* = 3. (**C** and **D**) The Lsm-ring binding to U6 increases in *mex67-5*. (C) Western blot of the Lsm8–GFP co-IP for the RIP experiment. The mitochondrial protein Aco1 served as a negative control unspecific binding. (D) qPCR products of the snRNAs after RNA extraction from the RIP experiment from *mex67-5* cells compared to wild-type is shown; *n* = 4, *P < 0.05; **P < 0.01; ***P < 0.001. (**E** and **F**) Lsm8 binding to U4 and U6 decreases in *lhp1∆*. (E) Western blot of the Lsm8–GFP co-IP is shown. Aco1 served as a negative control. (F) Lsm8-co-immunoprecipitated RNAs were analyzed in qPCRs and values obtained from *LHP1* knock out strains were compared to that of wild-type cells; *n* = 3, *P < 0.05; **P < 0.01; ***P < 0.001. **(G)** The interaction between Lhp1 and Mex67 is disturbed in *lsm8-1* mutants. Western blot analysis of Lhp1-co-precipitated Mex67 is shown in the indicated strains. Aco1 served as a negative control. **(H)**  *LSM8* and *NPL3* genetically interact. 10-fold serial dilutions of the indicated single or double mutants were spotted onto YPD-full medium plates and incubated for 4 days at 25°C; *n* = 3. (**I** and **J**) Lhp1 binding to snRNAs is increased when nuclear degradation is inhibited. (I) Pulldown of GFP-tagged proteins was validated via western blot analysis. The co-precipitated Lhp1 was detected through its myc-tag. The mitochondrial protein Zwf1 served as a negative control for unspecific binding. (J) RIP-experiment with Lhp1 expressed in the *mtr4-G77D* mutant shifted to 37°C for 1 h and the result of a subsequent qPCR of the snRNAs is shown in comparison to wild-type; *n* = 3.

To investigate a potential quality control function for Lhp1 in the nucleus, we analyzed its interactions further. Lhp1 and the Lsm-ring bind to the 3′ end of pre-U6 [34, 35], where both proteins interact with each other (Fig. 2A and B, and Supplementary Fig. S2A and B). The Sm-ring was shown to be loaded onto U1, U2, U4, and U5 in the cytoplasm [6]. To investigate, whether the Lsm-ring is also attached to pre-U6 in the cytoplasm, we investigated the binding of its component Lsm8 to the snRNA in RIP-experiments. We compared the situation in wild-type to that in *mex67-5*, in which the nuclear export is blocked and found that the interaction between U6 and the Lsm-ring increased (Fig. 2C and D), suggesting that the Lsm-ring is loaded already in the nucleus, which is different to the Sm-ring loading to the RNAPII-produced snRNAs. Remarkably, the binding of the other snRNAs to the Lsm-ring rather decreased. This might reflect their, usually indirect, interaction at the spliceosome, which decreases when U6 is lacking.

*In vitro* experiments suggested that dissociation of Lhp1 from U6 may facilitate the increased binding of the Lsm-ring to U6 due to the Usb1-mediated processing [25]. To confirm this assumption, we did *in vivo* RIP experiments of Lsm8 and U6 in the *lhp1*∆ strain, in which we show that the interaction with pre-U6, but interestingly also with pre-U4 was decreased (Fig. 2E and F). This confirms a requirement for Lhp1 in the attachment of the Lsm-ring and implies that the di-snRNA might already be formed in the cytoplasm. As Lhp1 shows high homology to the other guard proteins, as well as genetic and physical interactions (Fig. 1), it is tempting to speculate that Lhp1 might be a surveillance factor for proper oligouridylation of pre-U6, that controls and maybe supports Lsm-ring attachment. Their joint binding might in turn allow the subsequent binding of Mex67, which would signal that this processing step was successful, and the RNA is ready to be exported. To investigate this experimentally, we carried out co-IPs of Mex67 with Lhp1 in wild-type and in the *lsm8-1* mutant, in which the Lsm-ring loading cannot occur [31]. We found that while the Mex67-Lhp1 interaction was clearly detectable in wild-type cells, it was reduced when the Lsm-ring could not be attached (Fig. 2G). These data suggest that Lhp1 can only bind to Mex67, when the Lsm-ring was successfully loaded, indicating that Lhp1 is indeed supporting the retention of immature pre-U6. Lsm-ring loading onto U6 and Lhp1-mediated Mex67 recruitment thus resembles a quality control step. This is further supported by a genetic interaction of *lsm8-1* with the guard protein knock out of *NPL3* (Fig. 2H). The synthetic lethality between these mutants might indicate that the loss of one surveillance step is tolerable, but not when another maturation step was faulty.

In case the interaction of the guard proteins Npl3, Gbp2, and Hrb1 with Mex67 is not successfully accomplished, it was shown earlier that they recruit degrading enzymes to defective mRNAs instead of the export receptor. Nuclear degradation occurs mostly via the TRAMP complex, which marks the faulty RNAs with an oligo(A) tail that is subsequently recognized and eliminated by the nuclear exosome. Interestingly, Lhp1 interacts with the TRAMP-complex and the nuclear exosome component Rrp6 (Fig. 2I) [36, 37], suggesting that it might work in a similar fashion as the other guard proteins using a switch-like mechanism to either promote export of correctly matured pre-U6 or to initiate degradation of faulty pre-U6. In support of such a model, we found that mutation of *MTR4* and thus the TRAMP-complex, resulted in a significant increased binding of Lhp1 to the snRNAs in RIP-experiments (Fig. 2J and Supplementary Fig. S2C), suggesting that Lhp1 awaits degradation of the faulty RNA and might possibly actively recruit Mtr4 and the TRAMP-complex to the defective snRNA, which is inhibited in *mtr4(G677D)*.

### The Lsm-ring interacts with the re-import factors Mtr10 and Cse1

**Figure 3.**
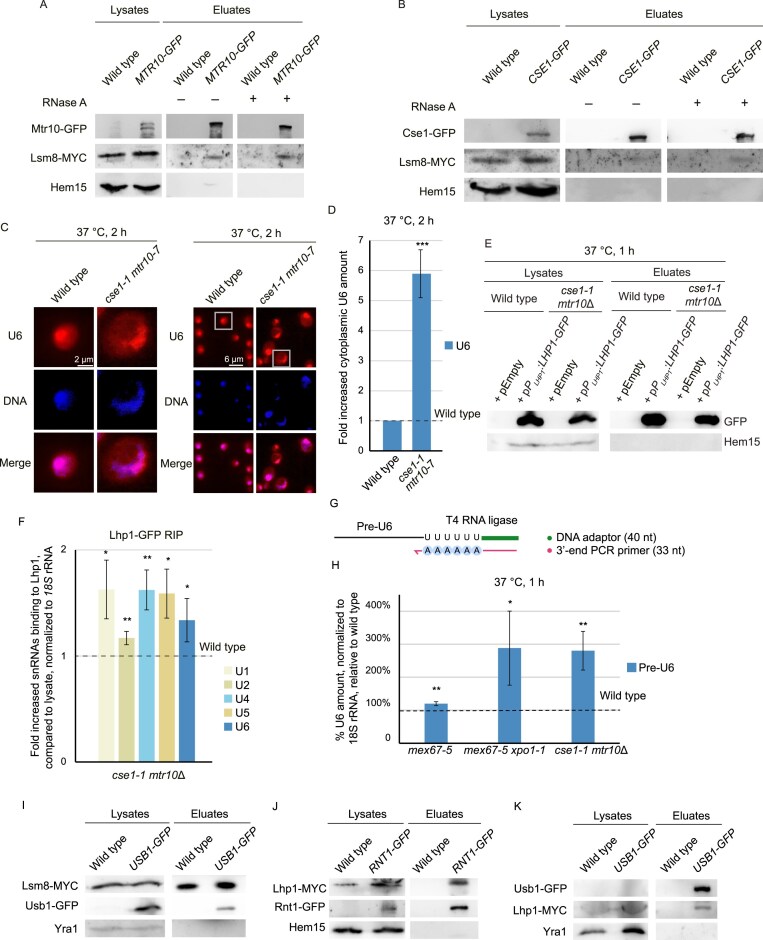
Lsm8 contacts the import factors Mtr10 and Cse1 in the presence of Lhp1. (**A** and **B**) The Lsm-ring physically interacts with Mtr10 and Cse1. (A) Western blot analysis of co-precipitated Lsm8-myc with Mtr10–GFP is shown. The monothiol protein Hem15 served as a negative control for unspecific binding; *n* = 3. (B) Western blot of a Cse1–GFP IP is shown with the co-precipitated Lsm8-myc; *n* = 3. (**C**) U6 accumulates in the *cse1-1 mtr10-1* double import mutant. FISH experiment with a Cy3-labelled specific probe targeting U6 is shown for the indicated strains that were shifted to the nonpermissive temperature for 2 h. DAPI was used to stain the nuclei. The exposure time of the Cy3 channel was 4s; *n* = 3. (**D**) Quantification of the cytoplasmic signal shown in (C) of at least 20 cells is depicted; *n* = 3. (**E** and **F**) Lhp1 remains bound to the pre-snRNAs after nuclear export. (E) Western blot analysis of the co-IP of Lhp1 is shown in the indicated strains. Hem15 served as a negative control. (F) qPCR result for the indicated snRNAs is shown after the Lhp1–GFP RIP experiment in the double import mutant compared to wild-type; *n* = 3, *P < 0.05; **P < 0.01; ***P < 0.001. (**G**) Scheme for the detection of the extended 3′ end of pre-U6 by 3′ end PCR. (**H**) An extended 3′ end of pre-U6 is detected in import and export mutants. 3′ end PCR was conducted from the RNA extracted from the indicated strains after a temperature shift to 37C for 1 h; *n* = 3; *P < 0.05; **P < 0.01; ***P < 0.001. (**I**) Physical interaction of Lsm8 with Usb1. Western blots of Lhp1 IPs are shown. The nuclear protein Yra1 served as a negative control. (**J** and **K**) Physical interaction of Lhp1 with Rnt1 (J) and Usb1 (K). Western blots of a Lhp1-myc IP (J) and a Usb1–GFP IPs (K) are shown. Hem15 and Yra1 served as washing controls, respectively.

Nuclear re-import of the RNAPII produced UsnRNAs is mediated by the Sm-ring that binds to the karyopherins Mtr10 and Cse1 [6, 18]. The RNAPIII generated U6, however, binds the Lsm2-8 ring. A mislocalization of both U6 and U4 was reported in *lsm8-1* mutants [6], suggesting that the Lsm-ring might participate in the nuclear re-import of these snRNAs. To investigate whether this ring is, like the Sm-ring, able to contact Mtr10 and Cse1, we carried out co-IP experiments with Lsm8. Even with the addition of RNase A we detected a physical interaction between the import receptors Mtr10 and Cse1 and Lsm8 (Fig. 3A and B), suggesting that the Lsm-ring might participate in the nuclear re-import of pre-U6. Similar results were obtained in *in situ* hybridization experiments with U6. Comparing its localization in wild-type to that of the double import mutant *cse1-1 mtr10-1* revealed a ∼6-fold increase of the cytoplasmic U6 signal, indicating re-import defects (Fig. 3C and D). Overall, the cytoplasmic mislocalization is not very strong, which might suggest that either the turn-over rate of U6 is so low that only few freshly made pre-U6 RNAs shuttle within the 2 h into the cytoplasm, or that other, currently unknown nuclear import receptors support re-import of pre-U6.

### Lhp1 remains bound to pre-U6 in the cytoplasm

*In vitro* experiments suggested that Lhp1 leaves pre-U6 after binding of Prp24, as Lhp1 shows a competitive binding with Prp24 *in vitro*, which is not the case for the Lsm-ring [25]. However, *in vivo* studies are missing and since Lhp1 turned out to function in the nuclear quality control and export of pre-U6, its immediate dissociation from the RNA upon Lsm-ring attachment before nuclear export can be excluded (Figs 1 and 2). To investigate whether Lhp1 dissociates in the cytoplasm, we performed RIP experiments and subsequent qPCRs in the double import mutant *cse1-1 mtr10∆* shifted to the non-permissive temperature for 1 h. Clearly, the binding of Lhp1 to all snRNAs increased (Fig. 3E and F), suggesting that the protein remains bound to pre-snRNAs after export. Interestingly, this experiment shows furthermore, that Lhp1 does not only control and support U6 maturation, but possibly also that of the other snRNAs. The binding of Lhp1 to U1, U2, U4, and U5 was shown earlier [31, 32]. However, a function on these RNAPII generated snRNAs has not been identified yet. As they do not contain an Lsm-ring, but an Sm-ring it is tempting to speculate that Lhp1 might surveil its attachment in the cytoplasm as well.

Pre-U6 is produced with a oligo(U) tail that is processed by the exonuclease Usb1 [38]. To find out if the trimming occurs before or after nucleo-cytoplasmic shuttling, we carried out 3′ end PCR of U6 in wild-type, in the export mutant *mex67-5 xpo1-1* and the re-import mutant *cse1-1 mtr10∆* and found increased amounts of the extended version, which is selectively amplified under the experimental conditions (Fig. 3G and H), suggesting that 3′ end processing occurs after shuttling. Trimming should stop at the bound Lsm-ring and indeed an interaction of Usb1 and Lsm8 was detected in co-IP experiments (Fig. 3I). As a guard protein Lhp1 might have its final surveillance function at this step and recruit the trimming enzymes Usb1 to pre-U6 and Rnt1 to pre-U4. To investigate this, we carried out co-IP experiments with Lhp1 and the two trimming enzymes and found that it interacts with both (Fig. 3J and K). In summary, we show that Lhp1 is recruited to all snRNAs in the nucleus. It remains bound to pre-U6 during the nuclear recruitment of the Lsm-ring and both Lhp1 and Lsm8 remain bound to pre-U6 in its cytoplasmic phase. Rnt1 and Usb1-mediated 3′ trimming occurs after shuttling in the nucleus and is bordered by the Lsm-ring.

### Prp24 supports U4/U6 annealing in the cytoplasm

As the Lsm-ring is loaded onto U6 already in the nucleus (Fig. 2C and D), which is different from the RNAPII UsnRNAs, onto which the Sm-ring is loaded within the cytoplasm [6], there seems to be no reason for U6 to shuttle out of the nucleus on the first sight. However, it was shown earlier that U6 and U4 accumulate in the cytoplasm of both *smb smd1* and *lsm8-1* mutants [6], suggesting that their re-import into the nucleus might be coupled and the di-snRNA formation might occur in the cytoplasm. However, formation of the U4/U6 di-snRNP requires Prp24 and since the protein is localized to the nucleus, it was anticipated that the di-snRNA is formed in this compartment.

**Figure 4.**
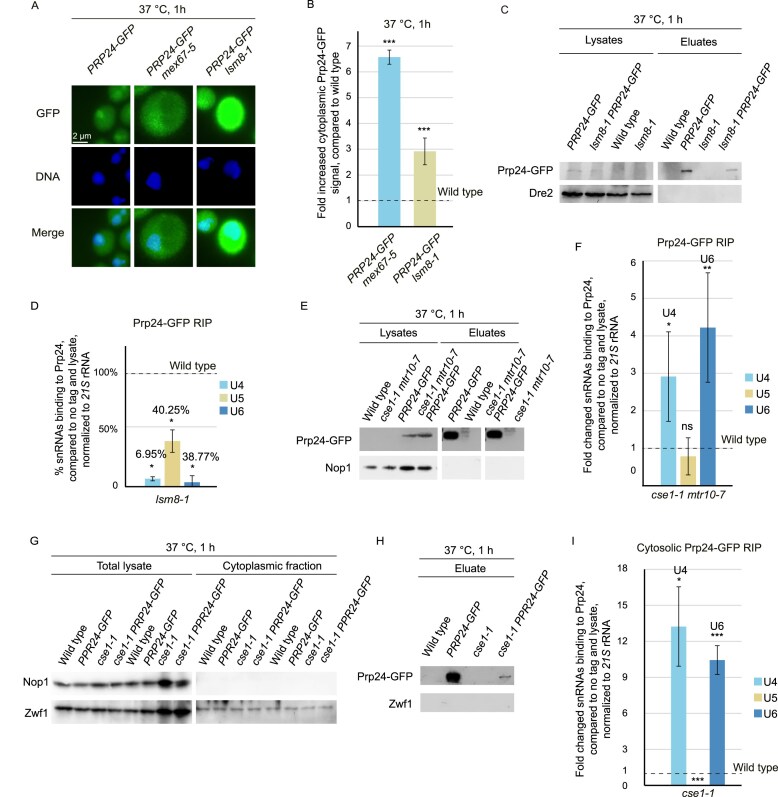
Prp24-mediated annealing of pre-U4 and pre-U6 occurs in the cytoplasm. (**A**) Prp24–GFP mislocalizes to the cytoplasm in the indicated mutant strains. Cells were grown to the log phase and shifted to the nonpermissive temperature for 1 h before the GFP-tagged Prp24 was monitored. The exposure time of the GFP channel was 4 s. (**B** and **C**) Prp24 binding to U4 and U6 decreases in *lsm8-1* mutant cells. (B) Quantification of the cytoplasmic signal shown in (A) of at least 20 cells per strain is depicted. (C) Western blot analysis of Prp24–GFP is shown in the indicated strains. The mitochondrial protein Dre2 served as a negative control for unspecific binding. (**D**) qPCR result of Prp24–RIP experiments from *lsm8-1* cells is shown and was compared to wild-type; *n* = 3, *P < 0.05; **P < 0.01; ***P < 0.001. (**E** and **F**) Prp24 binding to U4 and U6 increases when the re-import of U4 and U6 is blocked. (E) Western blot analysis of the Prp24 IP in the indicated strains is shown. The nucleolar protein Nop1 served as a washing control for unspecific binding. (F) qPCR result after a Prp24–RIP is shown in the double import mutant; *n* = 3, *P < 0.05; **P < 0.01; ***P < 0.001. (**G–I**) Nucleo-cytoplasmic fractionation RIP experiment (CRIP) reveals highly enriched binding of Prp24 to U4 and U6 in *cse1-1* cells. (G) Western blot analysis of the total lysate and the cytoplasmic fraction with the nuclear Nop1 and the cytoplasmic Zwf1 proteins. (H) Western blot of the Prp24-IP from the cytoplasmic fraction of the *cse1-1* strain. The cytoplasmic protein Zwf1 served as a negative control for unspecific binding. (I) qPCR result of the CRIP experiment with Prp24 in *cse1-1; n* = 3, *P < 0.05; **P < 0.01; ***P < 0.001.

To shed light into these opposing models, we analyzed whether Prp24 might be an exclusively nuclear protein, or if just its steady state localization is nuclear, because it spends more time in this compartment. Indeed, we found a primarily nuclear localization for Prp24–GFP (Fig. 4A and B, and Supplementary Fig. S3A–C). If the Prp24-mediated di-snRNP formation would occur in the cytoplasm, one would expect that blocking the export of the pre-snRNAs would lead to a re-location of Prp24 into the cytoplasm. Strikingly, a strong (∼6.5-fold increased) cytoplasmic localization of functionally GFP-tagged Prp24 was indeed detectable in the *mex67-5* mutant (Fig. 4A and B, and Supplementary Fig. S3A and B), suggesting that the protein is not imported into the nucleus, without being bound to pre-U6. To get an independent confirmation of the cytoplasmic binding of Prp24 to pre-U6, we analyzed its localization also in *lsm8-1* cells, in which the Lsm-ring is not attached [31]. As proper Lsm-ring binding to U6 should be a prerequisite for correct maturation and di-snRNA formation, we expected to find Prp24 mislocalized to the cytoplasm also in this situation, which was indeed the case (Fig. 4A and B, and Supplementary Fig. S3B). We find an approximately 3-fold increased cytoplasmic Prp24–GFP-signal in the cytoplasm. These findings support a model in which Prp24 binding to pre-U6 and di-snRNP-formation with pre-U4 occurs in the cytoplasm. However, these data do not allow to conclude whether Prp24 can bind to U6 in the absence of a proper Lsm-ring. Therefore, we carried out RIP experiments with Prp24 in the *lsm8-1* mutant. Subsequent qPCRs revealed a significantly decreased binding of the protein to U4 and U6 (Fig. 4C and D), indicating its disability to bind to the snRNAs without the Lsm-ring. After di-snRNA formation, the tri-snRNP composed of U4/U6/U5 usually forms and its reduced formation in *lsm8-1* is reflected by a decrease in the Prp24 interaction with U5. Finally, we confirmed the cytoplasmic attachment of Prp24 in the double import mutant *cse1-1 mtr10-7*, which in comparison to the *mtr10*∆ showed a stronger effect after induction of the mutation at 37°C. Prp24 RIP experiments and subsequent qPCRs in this strain resulted in an increased Prp24 binding to U4 and U6 (Fig. 4E and F). The binding of Prp24 to pre-U4 and pre-U6 were 3- and 4-fold increased, respectively. This reflects the average binding in the complete cell. However, to determine the binding of Prp24 and U4 and U6 only in the cytoplasm, we carried out a cytoplasmic fractionation experiment, in which the nuclei are eliminated and only the cytoplasmic fraction is analyzed further in subsequent RIP and qPCR experiments. As such an experiment is challenging, because the cells must be shifted to the nonpermissive temperature and at the same time treated with zymolyase, we realized that the double import mutant does not survive this procedure. Therefore, we used the *cse1-1* single mutant for this experiment. By this indirect *in vivo* approach for di-snRNA formation, we found that the binding of Prp24 to pre-U4 and U6 was even increased to about 10- to 12-fold (Fig. 4G–I). Together, these experiments confirm that the enzymatic activity of Prp24 is required in the cytoplasm to anneal pre-U4 with pre-U6 prior to nuclear re-import.

### Cis mutations that prevent U4/U6 annealing lead to cytoplasmic accumulation of U4 and U6

**Figure 5.**
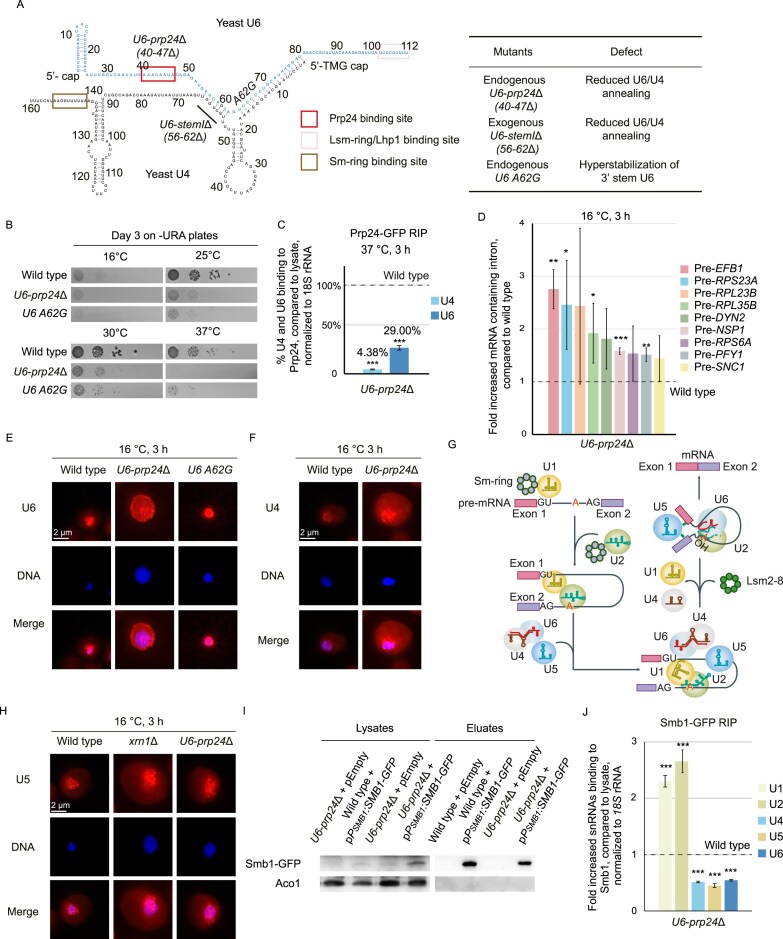
Mutation of U6 that prevent di-snRNP formation result in Prp24, U6, and U4 mislocalization. (**A**) Scheme of the U4/U6 di-snRNA with the created mutants of U6. (**B**) Growth analysis of the U6 mutants in 10-fold serial dilutions spotted onto -URA plates and incubated at the indicated temperatures for 3 days. (**C**) Binding of Prp24 to U4 and U6 is reduced in *U6-prp24∆*. qPCR results of the Prp24 RIP experiment in the *U6-prp24∆* mutant compared to wild-type; *n* = 4, *P < 0.05; **P < 0.01; ***P < 0.001. (**D**) Splicing is impaired in the *U6-prp24∆* mutant. qPCR results of the indicated genes amplified from the *U6-prp24∆* mutant compared to wild-type, *n* = 3, *P < 0.05; **P < 0.01; ***P < 0.001. (**E**) U6 localizes to the cytoplasm when the Prp24 binding region is mutated or the di-snRNA formation with U4 is impaired. FISH experiments with a Cy3-labeled-specific probe for U6 is shown in the indicated mutants, shifted for 3 h to 16°C. The exposure time of the Cy3 channel was 4 s for all shown *FISH* experiments. DAPI was used to stain the nuclei. (**F**) U4 accumulates in the cytoplasm of the *U6-prp24∆* mutant. FISH experiment with a Cy3-labeled U4-specific probe is shown after shifting the indicated strains to 16°C for 3 h. (**G**) Scheme for the stepwise subsequential U4/U6/U5 tri-snRNP formation. (**H**) Prevention of U4/U6 di-snRNA formation does not lead to a mislocalization of U5. FISH experiment with a Cy5-labeled U5-specific probe was carried out in the indicated strains after temperature shift to 16°C for 3 h. The DNA was stained with DAPI. (**I** and **J**) The binding of the Sm-ring to the snRNAs is altered when the di-snRNP is not formed. (I) Western blot analysis of an example Smb1–GFP IP for the RIP experiment shown in (J). The mitochondrial protein Aco1 served as a negative control for unspecific binding. (J) qPCR results from the Smb1 RIP experiment that was carried out in the *U6-prp24∆* mutant, compared to wild-type; *n* = 3, *P < 0.05; **P < 0.01; ***P < 0.001.

**Figure 6.**
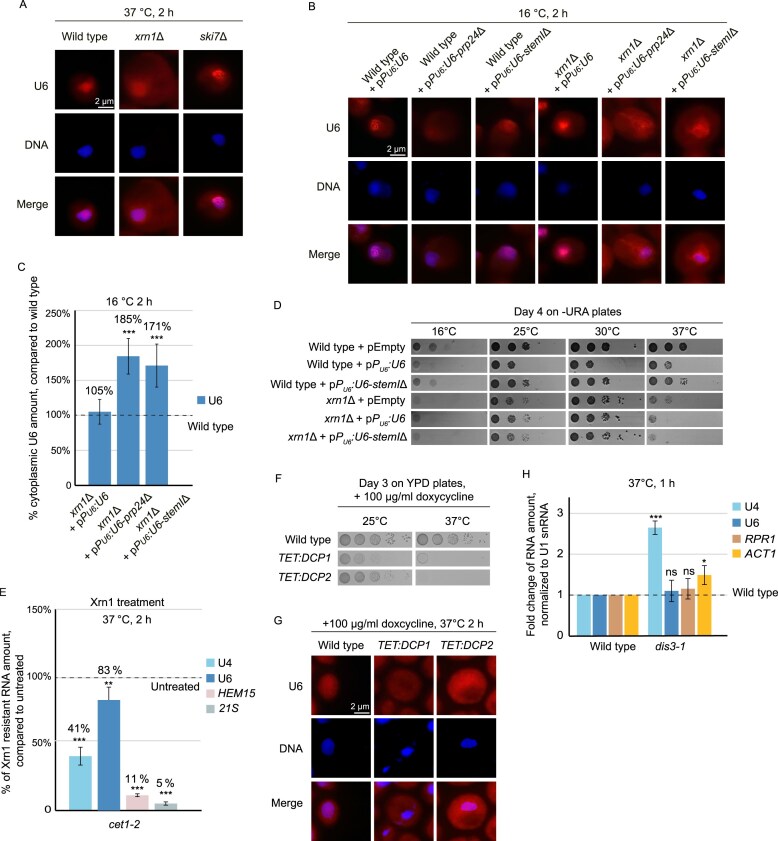
Faulty U4/U6-di snRNP formation is detected in the cytoplasm and defective U6 is eliminated from its 5′ end. (**A**) U6 accumulates in the cytoplasm of *xrn1*∆. FISH experiments with a Cy3-specific U6 probe was carried out in the indicated strains after a 2 h shift to the nonpermissive temperature of 37°C. The exposure time of the Cy3 channel was 4 s for all shown *FISH* experiments. (**B**) Overexpression of defective U6 in *xrn1∆* leads to an accumulation of faulty U6 in the cytoplasm. FISH experiments with a Cy3-labeled-specific U6 probe were carried out in the indicated strains that were shifted to 16°C for 2 h. DAPI was used to stain the nuclei; *n* = 3. (**C**) Quantification of the cells with U6 mislocalization phenotype shown in (B); *n* = 3, 25–30 cells were counted. (**D**) Overexpression of mutant U6 (*U6-stemI∆*) is toxic when expressed in *xrn1∆*. 10-fold serial dilutions were spotted onto selective agar plates and incubated at the indicated temperatures. (**E**) U6 is protected from Xrn1 digestion by a 5′ cap that is not an m^7^G-cap. RNA was isolated from wild-type and the *cet1-2* mutant after a temperature shift to 37°C for 2 h. Recombinant Xrn1 was incubated with the RNA for 2 h at 37°C and subsequent qPCR analysis was performed with the indicated genes; *n* = 3. (**F**) Downregulation of *DCP1* and *DCP2* results in cell death. 10-fold serial dilutions were spotted onto agar plates, containing doxycycline. (**G**) Dcp1 and Dcp2 are required for the degradation of U6 in the cytoplasm. FISH experiments with a Cy3-specific U6 probe were carried out in the cells containing either a tetracycline down-regulatable *DCP1* or *DCP2* gene, that were treated with doxycycline at the nonpermissive temperature for 2 h; *n* = 3. (**H**) qPCR experiments are shown for the indicated strains that were shifted to 37°C prior to cell lysis and RNA extraction. The target RNAs are indicated and *RRP1* and *ACT1* represent a ncRNA and mRNA, respectively, as controls; *n* = 3.

Binding of Prp24 to U6 was determined to occur at position 40 to 58 (Fig. 5A) [39]. To prevent/or reduce Prp24 binding, we created a partial genomic deletion of the nucleotides 40–47 (Fig. 5A). This partial deletion *U6-prp24∆ *[40–47], short *U6-prp24∆* still contains the CAGAGA box, which is important for the recognition of the 5′ splice site [39]. Two other mutations in U6 were created: *U6-A62G* that was shown to display a U4–U6 assembly defect, resulting in a growth defect and thereby increasing the proportion of free U4 and U6 (Fig. 5B) [40] and a *U6-stemI* deletion mutant *U6-stemI∆*[56–62] short U6 that had to be expressed from a plasmid over the wild-type gene, as this mutation is lethal [41] but tolerable in the presence of wild-type U6 (Fig. 6D).

Since a knockout of *PRP24* is lethal, but the *U6-prp24∆* strain grows slightly at 25°C (Fig. 5B), we assumed that the binding of Prp24 is impaired but not completely lost in the *U6*-*prp24∆* strain. To analyze that, we carried out RIP experiments and subsequent qPCRs with Prp24–GFP. We found that the binding is indeed reduced to ∼18% in the presence of this mutation at the non-permissive temperature (Fig. 5C and Supplementary Fig. S4A). To investigate whether the reduced binding of Prp24 in the *U6-prp24∆* would be sufficient to cause splicing defects, we used several randomly chosen example genes and analyzed the presence of their intron sequences by qPCR with specific primers in wild-type and *U6-prp24∆* (Supplementary Fig. S4B). The intron sequences were indeed increased between 1.5 and around 3-fold (Fig. 5D), which confirm splicing defects. Most importantly, we finally asked, in which compartment U4 and U6 are localized in the binding defective U6-mutants. We did the experiment at 16°C, because it was noted that the annealing between U4 and U6 is elevated at higher temperatures [42]. While both U4 and U6 were nuclear in the assembly mutant *U6(A62G)*, they localize to the cytoplasm in *U6-prp24∆* (Fig. 5E and F, and Supplementary Fig. S4C and D), confirming that the di-snRNA cannot be properly formed and partially paired and/or unpaired RNAs remain within the cytoplasm. Interestingly, the binding of the Lhp1 protein to U4 in *U6-prp24∆* significantly increased, while binding of Lhp1 to *U6-prp24∆* was unaltered compared to wild-type as shown in RIP experiments (Supplementary Fig. S4E and F). This could reflect that some U4 is re-imported into the nucleus like the other RNAPII snRNAs when not captured by U6 in the cytoplasm. Furthermore, as Lhp1 remains bound to U4 in the presence of *U6-prp24*∆, even after its cytoplasmic escape in *U6-prp24∆*, we suggest that Lhp1 might quality control the formation of duplex of U4 and U6 and protect intact U4 from degradation. The interaction of Lhp1 with the trimming enzymes might support a model in which it recruits the enzymes for final trimming and quality control of it.

### The tri-snRNA U4/U6/U5 is formed in the nucleus

After successful U4/U6 di-snRNP formation, U5 joins the complex to build the tri-snRNP, which subsequently enters the splicing complex (Fig. 5G). To investigate whether the tri-snRNP is formed in the nucleus upon entry of the U4/U6 di-snRNP, we analyzed the localization of U5 in the *U6-prp24∆* mutant and found that it is not mislocalized to the cytoplasm (Fig. 5H and Supplementary Fig. S4G). This indicates that U5 shuttles back into the nucleus after its Sm-ring recruitment in the cytoplasm [6], independently of the di-snRNP formation. This further suggests that the tri-snRNP, necessary for ongoing splicing would not be formed in this situation. Thus, the Sm-ring, which usually remains bound to all snRNAs during the snRNP assembly required for efficient trimethylation [43] (Fig. 5G), would remain bound to U1 and U2, as they would be retained at the 5′ splice site and the branch point sequence. Subsequent release of the components might be prevented in this situation. To investigate whether the Sm-ring is still bound to U1 and U2 when the U4/U6 di-snRNP is not formed, RIP experiments with Smb1 of the Sm-ring were carried out in the *U6-prp24∆* mutant and an increased binding of Smb1 to U1 and U2 could be shown (Fig. 5I and J). The increased Sm-ring binding to U1 and U2 in *U6-prp24*∆ supports a view that the splicing reaction arrests due to the lack of the U4/U6 di-snRNP and in this way U1 and U2 escape degradation, while U4, U5, and U6 are rather instable as they are not incorporated into the spliceosome. Together, these data suggest a model in which U5 is re-imported into the nucleus before U4/U6/U5-tri snRNP formation.

### Impaired U4/U6 di-snRNP formation in the cytoplasm leads to 5′ decay of pre-U6

A big advantage of the compartmental maturation of the U4/U6 di-snRNP is that defects in this process can be identified and faulty RNAs can be eliminated before they would come in contact with the spliceosome and inhibit pre-mRNA splicing. To investigate their potential involvement in the degradation of unpaired U4 and U6 snRNAs, we visualized the localization of U6 in mutants of the cytoplasmic degradation machinery. Interestingly, while we did not see any accumulation of U6 in a mutant of the 3′ end degrading Ski-complex, *ski7∆*, the deletion of the 5′ exonuclease *XRN1* resulted in a cytoplasmic signal for U6 (Fig. 6A and Supplementary Fig. S5A), suggesting that the elimination of faulty and unpaired U6 snRNAs takes place from their 5′ ends. To challenge the cytoplasmic quality control mechanism and produce less intact U4/U6 di-snRNPs, we expressed the U4/U6-annealing defective mutant *U6-stemI∆* and expressed it in wild-type and in *xrn1*∆. In the presence of an intact Xrn1, no significant change was visible. However, when the 5′ end degradation could not properly occur, we found an almost 2-fold increased cytoplasmic signal for U6 (Fig. 6B and C, and Supplementary Fig. S5B), confirming their cytoplasmic degradation from the 5′ end via Xrn1. Growth analysis revealed that the overexpression of U6 slightly affects growth, which indicates the importance of a balanced level of this snRNA. However, while the overexpression of the *U6-stemI*∆ mutant was tolerated in wild-type cells and did not lead to growth defects, this overexpression was toxic in *xrn1*∆ and resulted in a growth defect at 37°C (Fig. 6D).

Xrn1 can degrade uncapped mRNAs from their 5′ ends and mitochondrial *21S* rRNA with a nictotinamide adenine dinucleotide cap (NAD-cap) [44, 45]. It is currently unknown, whether U6 is capped at its 5′ end and whether the potential cap needs to be eliminated before the RNA can be degraded by Xrn1. Therefore, we investigated whether U6, which we purified either from wild-type yeast cells or from the mutant strain *cet1-2*, both shifted to 37°C, would be degraded *in vitro* by recombinant Xrn1. In the *cet1-2* stain, m^7^G-capping does not occur at the non-permissive temperature as it expresses a defective 5′ triphosphatase and much of the mRNAs and the U4 snRNA is uncapped [10]. The isolated RNA from both strains was incubated with recombinant Xrn1 *in vitro* for 2 h and subsequent qPCRs revealed that Xrn1 was able to degrade U4, the mRNA *HEM15*, and the mitochondrial *21S* rRNA, which was shown to be Xrn1 sensitive [45], but not U6 (Fig. 6E). Over 80% of U6 was present after the Xrn1 incubation, which indicates that pre-U6 contains a protecting 5′ cap, that is not an m^7^G-cap, or that the stable 5′stem loop structure prevents Xrn1 access [46]. To confirm *in vivo* that de-capping needs to take place before Xrn1 can act on pre-U6, we investigated its localization in mutants lacking the de-capping factors. Since both genes are essential, we downregulated their expression with a *TET:off* promoter. Addition of doxycycline to the strains resulted in cell death (Fig. 6F). Therefore, we added the drug for 2 h to liquid cultures and analyzed the U6 localization in FISH experiments. Clearly, the overall nuclear localization of U6 changed to a mainly cytoplasmic localization in both *DCP1* and *DCP2* knock down strains (Fig. 6G and Supplementary Fig. S5C). To confirm that the degradation of faulty pre-U6 indeed occurs from its 5′ end and not its 3′ end was confirmed by qPCR analysis of U6 in the 3′ end degrading exosome mutant *dis3-1*, in which U6 level did not change (Fig. 6H). Taken together, these results suggests that the formation of the di U4/U6 snRNA is monitored in the cytoplasm and in case it is not formed, faulty U6 is eliminated in the cytoplasm from their 5′ ends by the successive action of Dcp1- and Dcp2-mediated de-capping and degradation by Xrn1.

### A guard protein mediated quality control mechanism retains faulty pre-U6 in the cytoplasm

A switch like mechanism was suggested to regulate nuclear quality control, in which the guard proteins would bind Mex67 for nuclear export on correct mRNAs, but not on faulty RNAs, where they rather recruit the nuclear degradation machinery [7, 10, 13, 14]. A similar mechanism is conceivable for the re-import of the U4/U6 di-snRNP. When guard proteins remain bound on U4 and U6, their persistent binding might lead to the inevitable final recruitment of the cytoplasmic degradation machineries.

**Figure 7.**
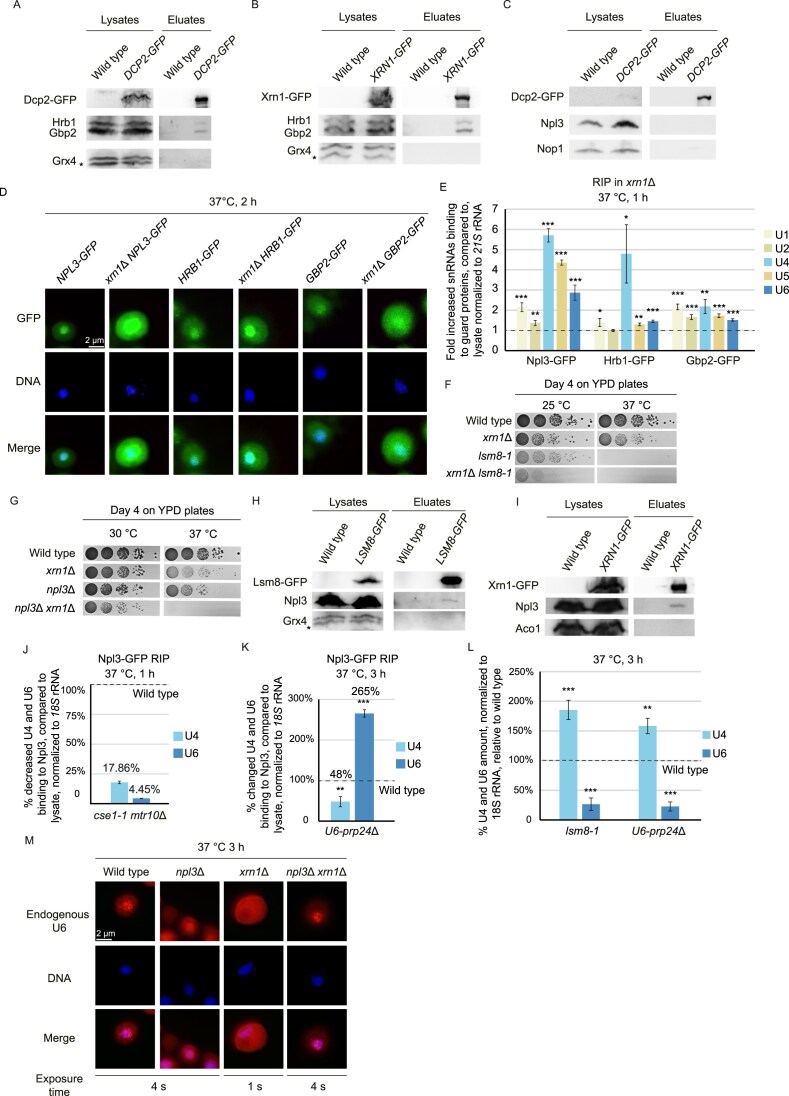
The guard proteins surveil annealing of U4 and U6 and mediate the degradation of faulty U6. (**A–C**) The guard proteins physically interact with the cytoplasmic 5′ degradation machinery. (A) Western blot analysis of the Hrb1 and Gbp2 co-IP with Dcp2–GFP is shown. The monothiol protein Grx4 served as washing controls for unspecific binding The double band for Grx4 shows proper washing of the eluates; *n* = 3. * indicates a degradation product of Grx4. (B) Western blot analysis of the Hrb1 and Gbp2 co-IP with Xrn1–GFP is shown. Grx4 served as a negative control; *n* = 3. (C) Western blot analysis of the Npl3 co-IP with Dcp2–GFP is shown. The nucleolar protein Nop1 served as a washing control for unspecific binding; *n* = 3. (**D**) The guard proteins Npl3, Gbp2, and Hrb1 accumulate in the cytoplasm of *xrn1∆*. GFP-tagged proteins were localized in the indicated strains after a 2 h temperature shift to 37°C. The exposure time of the Cy3 channel was 4 s. The DNA was stained with DAPI; *n* = 3. (**E**) Guard protein binding is enhanced in *xrn1∆*. RIP experiments with the indicated GFP-tagged guard proteins were carried out to detect the bound snRNAs; *n* = 3, *P < 0.05; **P < 0.01; ***P < 0.001. (**F**) Combined deletion of *XRN1* and *LSM8* have strong growth defects. 10-fold serial dilutions of the indicated mutant strains are shown on full medium YPD plates that were incubated at the indicated temperatures for 4 days. (**G**) Npl3 and Xrn1 are synthetically lethal. 10-fold serial dilutions of the indicated strains are shown on full medium YPD plates that were incubated at the indicated temperatures for 4 days. (**H**) Npl3 interacts with the Lsm-ring. Western blot analysis of the Npl3 co-IP with Lsm8–GFP is shown. Grx4 served as a negative control for unspecific binding. (**I**) Physical interaction of Xrn1 and Npl3. Western blot analysis of the Npl3 co-IP with Xrn1–GFP is shown. Aco1 served as a negative control for unspecific binding. **(J)** Npl3 dissociates from U4 and U6 before nuclear re-import. Npl3 RIP experiment in the double import mutant *cse1-1 mtr10∆* was carried out from the isolated RNA collected after a temperature shift to 37°C for 1 h; *n* = 3. *P < 0.05; **P < 0.01; ***P < 0.001. (**K**) Npl3 binding to defective U6 is increased. Npl3 RIP experiment in the *U6-prp24∆* mutant was carried out from the isolated RNA collected after a temperature shift to 37°C for 3 h; *n* = 3; *P < 0.05; **P < 0.01; ***P < 0.001. (**L**) qPCRs of the isolated RNA from the indicated strains shifted to 37°C for 3 h reveals the changed U4 and U6 RNA levels; *n* = 3, *P < 0.05; **P < 0.01; ***P < 0.001. (**M**) The nuclear retention of faulty U6 is released in the absence of Npl3. FISH experiments with a Cy3-labeled-specific U6 probe were carried out in the indicated strains that were shifted to 37°C for 3 h. DAPI was used to stain the nuclei; *n* = 3.

The mRNA guard proteins Npl3, Gbp2 and Hrb1 were, in addition to their function as mRNA guards, also shown to bind to the pre-snRNAs and shuttle with them into the cytoplasm [6]. Moreover, in particular Gbp2 and Hrb1 were shown to participate in the cytoplasmic nonsense-mediated mRNA decay (NMD) by recruiting degrading enzymes to pre-mature stop-codon containing mRNAs [33]. Degradation of such faulty mRNA transcripts occurs from the 5′ end via Dcp1–Dcp2 mediated de-capping and subsequent 5′-3′ degradation via Xrn1 and by the Ski-complex mediated exosomal degradation from the 3′ end. A physical interaction between Gbp2 and Hrb1 with Dcp1 have been shown earlier [33]. Additionally, we could show a physical contact of Hrb1 and Gbp2 with Dcp2 and Xrn1 in co-IP studies. However, no such physical interaction was observed between Npl3 and Dcp2 (Fig. 7A–C and Supplementary Fig. S6A–C). To support a possible involvement of the guard proteins in the elimination of faulty U6, we analyzed if these, at steady state nuclear proteins, would accumulate in the cytoplasm of *xrn1∆* cells, in which potentially defective U6 accumulates. Indeed, all three proteins were increasingly detected within the cytoplasm of *xrn1*∆ (Fig. 7D and Supplementary Fig. S6D). In this situation we show that the interaction of the guard proteins with the snRNAs is increased, in particular for Npl3 (Fig. 7E, and Supplementary Fig. S6E and F), which could reflect that they retain them in the cytoplasm until the faulty RNA is degraded.

Moreover, genetic interactions with each other, the Lsm-ring and *xrn1*∆ were detected (Figs 1H and 7F, G, and Supplementary Fig. S6G, H), underlining their related functions and supporting a possible cytoplasmic function for Npl3 in RNA degradation. Its function on pre-U6 is further supported by its physical contact with the Lsm-ring and the cytoplasmic Xrn1 as shown in co-IPs (Fig. 7H and I, and Supplementary Fig. S6I and J).

Taken together our data suggest that Npl3 in support of Gbp2 and Hrb1 might retain faulty RNAs from being reimported into the nucleus. If this is the case one would expect that the interaction of Npl3 would decrease in an import mutant, because all snRNAs are present in a matured and correct form. Indeed, as shown by RIP experiments, the binding of Npl3 to U4 and U6 is significantly reduced (Fig. 7J and Supplementary Fig. S6K), reflecting that they dissociated. Importantly, this is not the case when U4 and U6 cannot anneal. In the *U6-prp24∆* mutant we get a strong increase of Npl3 binding to U6 (Fig. 7K and Supplementary Fig. S4A). Strikingly, the opposite was seen for U4, which might indicate that it might not be retained in the cytoplasm when the guard proteins dissociated and U6 is not available. In fact, qPCRs revealed that while the U6 level decreased in both *lsm8-1* and *U6-prp24∆* mutants, in which the di-snRNP cannot be formed, the level of U4 rather increased (Fig. 7L), suggesting that it might be reimported into the nucleus alone, without being bound by U6 as the other snRNA. However, as it cannot be incorporated into the spliceosome, it rather accumulates. An exclusive nuclear localization of U4, however, was not detected in the *U6-prp24∆* mutant, possibly because of a partial binding of U4 to U6 (Fig. 5F).

**Figure 8.**
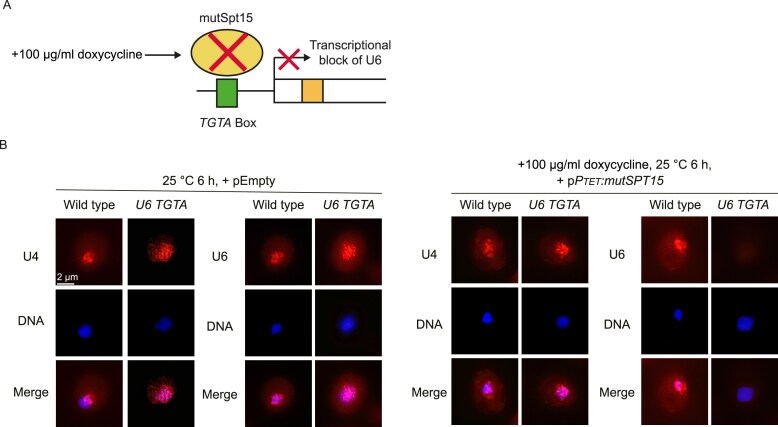
Specific downregulation of U6 prevents the cytoplasmic retention of U4. (**A**) Scheme of the experimental setup to downregulate the plasmid encoded U6 from the altered TATA-box (TGTA) with mutSpt15 adapted from [48]. (**B**) *In situ* hybridization of U4 and U6 in the indicated strains with specific Cy3-labeled probes. The exposure time of the Cy3 channel was 4 s. The DNA was stained with DAPI.

To finally proof a guard protein mediated retention mechanism of defective U6, we wanted to show a leakage of defective U6 from the cytoplasm into the nucleus in the absence of the retention factor Npl3. Therefore, we deleted *NPL3* in *xrn1*∆, in which much of the defective U6 accumulates, and localized U6. Strikingly, we found that the cytoplasmic retention was released and U6 entered the nucleus (Fig. 7M and Supplementary Fig. S6L), visualizing the cytoplasmic guard protein mediated snRNA quality control. To specifically downregulate *U6* to localize U4 in its absence we had to use a different strategy. Since a *U6* knock out is lethal to cells [47], we used a system that can selectively downregulate specific RNAPIII genes through a inducible mutated TATA-binding protein (mutSpt15) [48]. Upon the addition of doxycycline, when we block U6 transcription, U4 localizes exclusively to the nucleus (Fig. 8A and B, and Supplementary Fig. S7), indicating that it is only retained in the cytoplasm when U6 is expressed.

**Figure 9.**
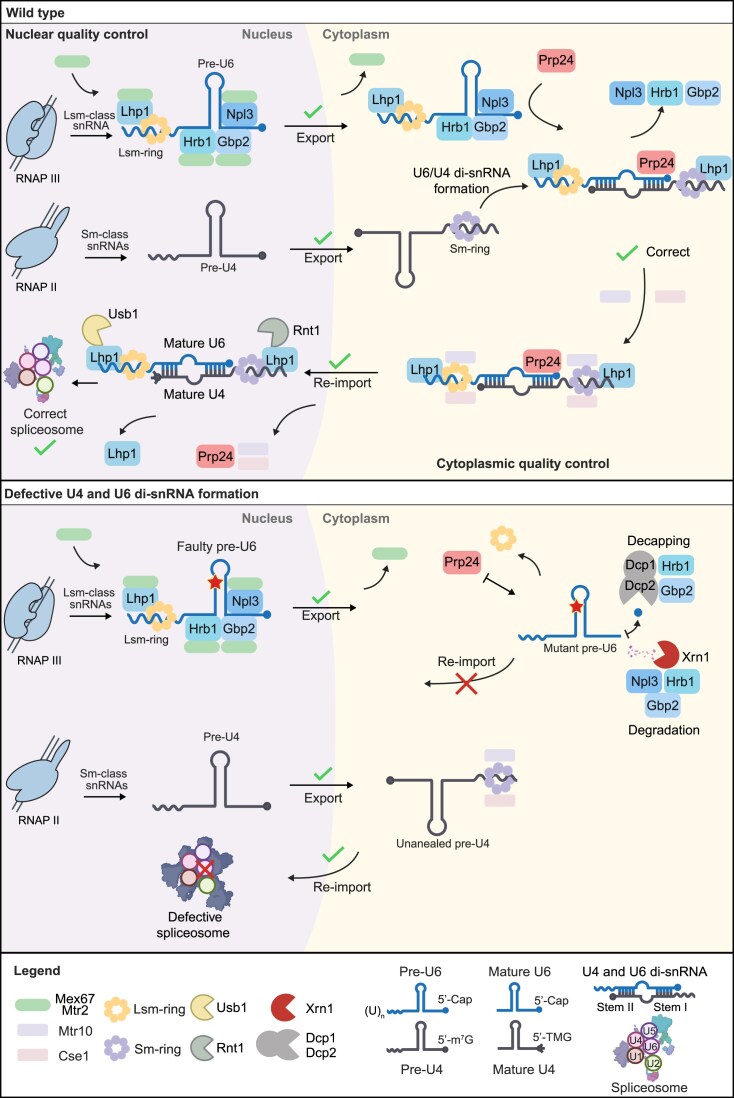
Model for the cytoplasmic quality control of the U4/U6 di-snRNA formation. Top: Pre-U4 is transcribed by RNAPII and is exported into the cytoplasm, where it receives its Sm-ring. Pre-U6 is generated by RNAPIII with a 3′ poly(U) tract to which Lhp1 binds. Subsequent loading of the Lsm-ring in the nucleus occurs. Lsm-ring attachment is recognized by the guard protein Lhp1, which subsequently recruits the export receptor Mex67–Mtr2 (short Mex67) to pre-U6. The other guard proteins, Npl3, Gbp2, and Hrb1, bind to pre-U6 as well and capture further Mex67 molecules, resulting in the nuclear export of the snRNA. In the cytoplasm Mex67 is displaced and Prp24 attaches and mediates the U4/U6 annealing, which results in the dissociation of the guard proteins Npl3, Gbp2, and Hrb1. Interaction of the import receptors Cse1 and Mtr10 with the Sm- and Lsm-rings of the di-snRNA elicit nuclear re-import. Final maturation steps include the Rnt1 and Usb1-mediated 3′ trimming up to the Sm- and Lsm-ring, respectively, which might be recruited by Lhp1 surveilling trimming. The mature U4/U6 di-snRNA is incorporated into the spliceosome. Bottom: In case a mutated pre-U6 is expressed and/or the annealing to U4 is otherwise disturbed, the guard proteins Npl3, Gbp2, and Hrb1 remain bound on the ssRNAs and recruit the de-capping factors Dcp1, Dcp2, and the exonuclease Xrn1 for degradation from the 5′ end of the snRNA. Pre-U4 is not retained in the absence of U6 and is re-imported into the nucleus, however, unable to support splicing without U6. Faulty U6 is prevented from re-import through association of Npl3. This guard protein leaves U6 upon annealing of U4 and remains bound if this fails, preventing its re-import into the nucleus until the defective U6 is degraded.

In summary, our results show that U4/U6 di-snRNP formation occurs in the cytoplasm by Prp24. We suggest a model in which snRNA maturation and proper di-snRNP formation is controlled by the guard proteins Npl3, Gbp2, and Hrb1 and the novel guard member Lhp1 (Fig. 9). They are loaded in the nucleus, control nuclear maturation steps such as 5′ capping, and Lsm-ring loading, before they recruit the export receptor Mex67. They shuttle with the snRNAs into the cytoplasm where the Sm-ring loading occurs, to enable pre-U1, -U2, and -U5 to re-enter the nucleus. Pre-U4 is prevented from re-import, because it is captured by the Prp24-mediated di-snRNA formation of pre-U4 and pre-U6. The cytoplasmic retention of the U4/U6 di-snRNA is relieved through dissociation of the guard proteins. Both, the Sm- and the Lsm-ring are subsequently able to interact with the nuclear re-import factors Cse1 and Mtr10, allowing their nuclear re-entry. In case the di-snRNA formation fails, as the case in the *U6-prp24∆* mutant, the guard proteins do not dissociate but recruit the cytoplasmic degradation machinery instead. This guard protein mediated quality control mechanism, which uses the different compartments for maturation and quality control, protects the cell from immature snRNAs that could torpedo splicing.

## Discussion

mRNA quality control occurs in the nucleus, where capping, splicing, cleavage, and polyadenylation is controlled and, in the cytoplasm, where the open reading frame is monitored. All surveillance steps involve the guard proteins, Npl3, Gbp2, Hrb1, Hrp1, and Nab2 [7, 9–11, 13, 14]. In this case the compartmental separation prevents the translation of intron containing pre-mRNAs, and thus the expression of e.g. truncated proteins. Little is known about the quality control of ncRNAs. Four snRNAs, U1, U2, U4, and U5 are, like mRNAs, synthesized by RNAPII and receive e.g. an m^7^G-cap. They bind the guard proteins [6] and it is likely, that their capping is surveilled in a similar manner as mRNAs. Like mRNAs, these snRNAs also exit the nucleus, however, not for translation but for maturation and association of the Sm-ring. This ring contacts the import receptors Cse1 and Mtr10 enabling their nuclear re-import [18]. Thus, nuclear re-import resembles a quality checkpoint for these snRNAs. Only after re-import, final trimming of the pre-snRNAs occur to form the mature snRNAs that enter the spliceosome [18]. Thus, maturation of these ncRNAs in different compartments is important to build an intact molecular machine for splicing. Such mechanism is related to the compartmental maturation of the telomerase, a ribozyme that contains an RNA, *TLC1*, which resembles a scaffold and a template for the elongation of the telomer ends on chromatin. Pre-*TLC1* is generated like snRNAs by RNAPII, exported into the cytoplasm via Mex67 and upon assembly of some core proteins and the Sm-ring Pre-*TLC1* is re-imported via Cse1 and Mtr10 [18, 49].

The snRNA U6 differs from the other snRNAs as it is transcribed by RNAPIII. In contrast to human U6, which is thought to never leave the nucleus, yeast U6 was shown to shuttle into the cytoplasm [6]. However, due to RNAPIII transcription it lacks the typical features of the other snRNAs and mRNAs, such as the m^7^G-cap, which is the docking site for CBC and the export factor Xpo1. However, as it requires de-capping before it can be degraded from its 5′ end, we suggest that it contains a 5′ cap structure, which is not an m^7^G-cap (Fig. 6E) as it is also not transported via the CBC-contacting Xpo1 [6]. Human U6 was shown to interact with an γ-monomethylguanosine triphosphate (meGTP)-antibody, suggesting that the γ-phosphate of the 5′ guanosine triphosphate is methylated [50]; however, the exact cap structure remains to be determined. In contrast to RNAPII transcripts, RNAPIII produced RNAs contain oligo(U) 3′ ends of variable lengths, ending with a *cis*-diol (2′,3′ hydroxyl group), important for protein assembly [23]. The oligo(U) stretch is recognized and bound by Lhp1 (human La), which was shown to stabilize newly synthesized snRNAs from degradation [31, 32]. We suggest, that Lhp1 protects correct pre-U6 RNAs in its function as a guard protein, because (i) it is highly homologues to the other guard proteins (Fig. 1D and Supplementary Fig. 1C); (ii) it interacts with Npl3, Gbp2, and Hrb1, typical for all guard proteins (Fig. 1E–G) [33]; (iii) it shows a genetic interaction with *npl3∆* (Fig. 1H); and (iv) it interacts with the export receptor Mex67 (Fig. 1A), very similar to the other guard proteins. Correct processing steps are flagged on mRNAs with this “export ticket” [7]. In contrast, upon defects in RNA processing the guard proteins prevent Mex67 binding and rather recruit different RNA degradation machineries. In fact, Lhp1 interacts with the TRAMP-complex and the nuclear exosome [36], further supporting its categorization as a guard protein. Generally, they work in a switch like mode to promote either export through Mex67 association or degradation of incorrect RNAs by capturing degradation factors [7]. To identify the special surveillance task for Lhp1, we did several key experiments with Lsm8 of the Lsm-ring. Lsm-ring binding was shown to be supported by Lhp1 [51] and (Fig. 2E and F). Furthermore, we have shown that the Lsm-ring, in contrast to the Sm-ring, is loaded onto the snRNA in the nucleus (Fig. 2C and D). Most importantly, our experiments show that the binding of Mex67 to Lhp1 is reduced, when the Lsm-ring was not properly loaded onto pre-U6 (Fig. 2G), revealing the quality control function of the novel guard protein Lhp1. Proper Lsm-ring loading is essential, because it supports the nuclear re-import of pre-U6 after shuttling (Fig. 3). *In vitro* studies have shown that Lhp1 preferentially binds a *cis*-diol rather than a 3′ phosphate, whereas the Lsm-ring, prefers a 3′ phosphate over a *cis*-diol. Thus, it was suggested that the exchange of the *cis*-diol to a 3′ phosphate might remove the binding of Lhp1 and increase the binding affinity of the Lsm-ring to U6 [25]. Generally, these findings agree with our results, because the order of binding, Lhp1 first and then the Lsm-ring, is supported by our data (Fig. 2F). However, our *in vivo* results moreover reveal that the dissociation of Lhp1 is not immediate. The interaction of both proteins with pre-U6 remains also in the cytoplasm, as shown for Lhp1 in the double import mutant *cse1-1 mtr10∆* (Fig. 3E and F). The Lsm-ring is only released in the nucleus, when the snRNP enters the spliceosome [43]. We suspect that Lhp1 has a second surveillance function, which is not restricted to U6 but also concerns the other snRNAs, as Lhp1 was also shown to bind to the other snRNAs [32]. Their Sm-ring loading, which occurs in the cytoplasm, and the continuing Lsm-ring attachment to U6 needs to be controlled, as the rings are prerequisite for nuclear re-import. Lhp1 on defective snRNAs persist, which supports a function in retention of defective snRNAs (Fig. 2J). In our model (Fig. 9), we therefore suggest that Lhp1 might dissociate only upon completion of the shuttling cycle upon recruitment of the 3′ trimming enzymes for pre-U6 and pre-U4, Usb1 and Rnt1, respectively (Fig. 3I–K) [6]. In accordance with its guard protein function, Lhp1 might recruit these degrading enzymes after shuttling for the final processing of the pre-snRNAs into mature components of the spliceosome.

Our study furthermore reveals that the di-snRNA formation of immature pre-U4/pre-U6, before trimming, always anticipated to occur in the nucleus [42, 47], takes place in the cytoplasm. We show that Prp24, responsible for U4 and U6 annealing, is a shuttling protein with an at steady state nuclear localization (Fig. 4A). However, interestingly, it accumulates in the cytoplasm when U6 is retained in the nucleus (Fig. 4A), indicating that Prp24 is waiting to be loaded onto U6 in the cytoplasm. In this situation Prp24 does not contact U6, while we showed an increased interaction of Prp24 and U6 in the import mutants (Fig. 4E–I). These data indicate that Prp24 activity to support di-snRNA formation is required in the cytoplasm and Prp24 shuttles with the U4/U6 di-snRNP into the nucleus, where it subsequently forms the U4/U6/U5 tri-snRNP (Fig. 5G and H).

We assumed that di-snRNP formation in the cytoplasm is also a quality-controlled maturation step for a functional spliceosome. To investigate this, we created several mutants to be able to produce enhanced amounts of defective pre-U6. Mutation of the Prp24 binding domain in U6 resulted in a slightly increased amount of pre-U6 in the cytoplasm, which was significantly enhanced when the 5′ exonuclease Xrn1, or the de-capping factors Dcp1 and Dcp2 were absent (Fig. 6A, B, and G), suggesting that pre-U6 undergoes surveillance and defective U6, which is not able to pair with U4, is retained in the cytoplasm until it is eliminated. Interestingly, pre-U4 is only retained in the presence of U6 but not in its absence (Figs 5E, F and 8A, B, and Supplementary Fig. S7). In the absence of the Prp24-binding site, U4 seems to be able to enter at least partially the nucleus, where it is protected from degradation leading to its accumulation (Fig. 7K and L). In the complete absence of U6, pre-U4 is not retained in the cytoplasm at all, which clearly shows that it needs U6 for its cytoplasmic retention (Fig. 8A and B, and Supplementary Fig. S7). These findings suggest that the U6 capturing dominates over nuclear re-import of unpaired U4.

Importantly, the di-snRNP formation is also controlled by guard proteins. Npl3, Gbp2, and Hrb1 interact with different degrading enzymes (Fig. 7A–C) [33] and the binding of Npl3 to mutant pre-U6 increases in *xrn1*∆ and *U6-prp24*∆ (Fig. 7E and K). Most importantly, the increased cytoplasmic presence of defective U6 in *xrn1∆* is relieved in the absence of Npl3 (Fig. 7M), identifying this guard protein as a cytoplasmic pre-U6 retention factor for faulty and unpaired U6.

In human cells, U1, U2, U4, and U5 shuttle also into the cytoplasm to receive the Sm-ring, like yeast. Their re-import occurs also through an import mechanism that involves the karyopherins to which also Mtr10 and Cse1 in yeast belong. The karyopherin in human cells is termed snurportin [52]. In contrast U6 is thought to remain nuclear in human [29, 30]; however, in yeast, we have shown that U6 also shuttles into the cytoplasm. However, the formation of the di-snRNP in a different compartment has the advantage for quality control and thus potential U6 shutting and cytoplasmic di-snRNP formation should be re-evaluated in human.

Taken together we suggest a novel model for the snRNA maturation cycle (Fig. 9). The data from [6] suggest that pre-snRNAs, transcribed by RNAPII, shuttle into the cytoplasm where they receive an Sm-ring, which enables them to shuttle back into the nucleus. In contrast, the RNAPIII transcript pre-U6 receives its Lsm-ring already in the nucleus. This is controlled by Lhp1, which has not only structural similarities but also functional similarities with the guard proteins Npl3, Gbp2, and Hrb1. They recruit the export receptor heterodimer Mex67–Mtr2 to the RNA for nuclear export. Upon export, Mex67 is displaced from pre-U6 and Prp24 associates. Its activity supports the formation of the U4/U6 di-snRNP, which prevents U4 from nuclear re-import on its own. Correct U4/U6 annealing leads to the association of the import receptors Cse1 and Mtr10 to the Sm- and Lsm-rings, which leads to nuclear re-import of the di-snRNA, bound to Prp24 and Lhp1. In the nucleus, Lhp1 might have a second role in quality control, where it interacts with Usb1 and maybe also Rnt1 to mediate trimming of U4 and U6, respectively. Lhp1 and Prp24 dissociate and the tri-snRNP, composed of U4/U6/U5 forms and participates in splicing.

In case of faulty U6 with which annealing to pre-U4 is not possible, U4 is not retained in the cytoplasm and shuttles back into the nucleus via the Sm-ring mediated import. Missing binding of U4, leads to the persistent binding of the guard proteins to pre-U6, that act as cytoplasmic retention factors and their extended binding leads to the recruitment of the de-capping machinery and Xrn1 to initiate the degradation of the faulty pre-U6.

Therefore, the compartmental assembly and the guard protein-mediated surveillance mechanisms prevent faulty di-snRNPs to torpedo the spliceosome, emphasizing the importance for the quality control of ncRNA. Additionally, this study illustrates that RNA surveillance mechanisms extend beyond coding RNAs and involve similar quality control mechanisms and proteins.

## Materials and methods

### Yeast strains, plasmids, and oligonucleotides

All *Saccharomyces cerevisiae* strains used in this study are listed in Supplementary Table S1, plasmids in Supplementary Table S2, and oligonucleotides in Supplementary Table S3. Strains were cultivated in standard media at 25 °C unless otherwise stated. Plasmids and yeast strains were generated by conventional methods. To knock out or mutate yeast strains, the regarding defective mutants were either crossed with each other, or generated by homologous recombination.

### Growth analysis of yeast strains

For drop dilution assays, the cells were grown to the logarithmic phase (2 × 10^7^ cells/ml). Subsequently, 10 μl of 10-fold serial dilutions with cell densities of 5 × 10^6^, 5 × 10^5^, 5 × 10^4^, 5 × 10^3^, and 5 × 10^2^ cells per ml were spotted onto agar plates. Cells were grown at the indicated temperatures for 2–4 days.

### Co-immunoprecipitation (IP) experiments

The experiments were conducted largely as previously described [6]. Cells were grown to mid log phase at 25°C and, if necessary, shifted to the indicated temperatures for the indicated times. Afterward, cells were harvested and lysed in PBSKMT buffer [1× PBS (137 mM NaCl, 2.7 mM KCl, 10 mM Na_2_HPO_4_, and 1.8 mM KH_2_PO_4_, pH 7.4), 3 mM KCl, 2.5 mM MgCl_2_, and 0.5 % Triton X-100, including protease inhibitors from Roche and Sigma] with glass beads using a FastPrep machine (MP Biomedicals) three times for 30 s at 6 m/s. 25 μl of the supernatant was taken for western blot analysis. The remaining supernatant was divided into two equal portions. RNase A was added to one portion at a final concentration of 200 μg/ml. GFP- or MYC-tagged proteins were precipitated with GFP- or MYC- conjugated beads (ChromoTek) for 5 h at 4°C. After incubation, the beads were washed five times with 1 ml PBSKMT buffer by gentle inversion. The lysate and eluate were analyzed on western blots with the indicated antibodies [GFP (Santa Cruz) 1:2000; Myc (9E10) antibody (Santa Cruz) 1:2000; Zwf1 (Sigma) 1:50 000; Hem15 (courtesy of R. Lill and U. Mühlenhoff, Marburg, Germany) 1:5000; Aco1 (courtesy of R. Lill and U. Mühlenhoff, Marburg, Germany) 1:2000; Mex67 (courtesy of C. Dargemont, Paris, France) 1:50 000; Gbp2 (self-made by David’s Biotechnology) 1:20 000; Hrb1 (self-made by David’s Biotechnology) 1:20 000; Tdh1 (Thermo Fisher Scientific) 1:5000; Grx4 (U. Mühlenhoff) 1:5000; Nop1 (courtesy of R. Lill and U. Mühlenhoff, Marburg, Germany) 1:5000; and Dre2 (courtesy of R. Lill and U. Mühlenhoff, Marburg, Germany) 1:4000. Finally, ECL substrate solution (WesternBright Quantum, Advansta) was applied to the membrane, and protein signals were detected using a chemiluminescence imaging device [Fusion FX7 Edge (Vilber)].

### RNA co-immunoprecipitation 

The RIP experiments were conducted largely as previously described [6]. All yeast strains were grown to mid log phase at 25°C. If necessary, cells were shifted to a nonpermissive temperature for the indicated time. Afterward, the cells were lysed in RIP buffer [25 mM Tris–HCl pH 7.5, 100 mM KCl, 0.25 % (v/v) Triton X-100, 0.2 mM phenylmethylsulfonyl fluoride (PMSF), 5 mM dithiothreitol (DTT), 0.02 U per ml RiboLock RNase Inhibitor (Thermo Scientific) and protease inhibitor (Roche)] with glass beads utilizing a FastPrep machine (MP Biomedicals) three times for 30 s at 6 m/s. 25 μl of the supernatant was taken for western blot analysis. GFP-trap beads or MYC-trap beads were used to precipitate GFP- or MYC-tagged proteins by incubating with the remaining supernatant for 2 h at 4°C. After incubation the beads were washed five times with 1 ml RIP buffer and split into two portions. rDNase (Qiagen) was added to one portion to eliminate the DNA contaminant for 30 min at 4°C and 15 min at 25°C for the subsequent RNA isolation via TRIzol-phenol-chloroform (Thermo Fisher Scientific) extraction, followed by cDNA synthesis (NIPPON Genetics) and detection of the snRNA level via qRT-PCR (Bio-Rad). The other portion of the eluate was analyzed by SDS–PAGE and subsequent western blot.

### Fluorescense *in situ* hybridization 

Fluorescense *in situ* hybridization (FISH) experiments were performed as reported earlier [53]. Cy3-labeled short (∼50 nt) sequence specific probes (Sigma) were used to stain nuclear snRNA. All yeast strains were grown to mid log phase at 25°C, and cells afterward were shifted to 16°C or 37°C for the indicated time. 10 ml of cells were fixed with 1 ml of deionized formaldehyde to a final concentration of 3.7 % for 15 min at the temperature to which the cells were shifted and the incubation was continued with agitation for 45 min at room temperature. Cells were washed and resuspended with P solution (1.2 M sorbitol and 0.1 M phosphate buffer, pH 6.5) and subsequently spheroplasted by adding zymolase (10 mg/ml, Zymo Research). Subsequently, cells were permeabilized by adding P solution containing 0.5 % Triton^®^X-100, and then prehybridized with Hybmix [50% deionized formamide, 5× SSC, 1× Denhardts, 500 µg/ml tRNA, 500 µg/ml salmon sperm DNA, 50 µg/ml heparin, 2.5 mM EDTA (pH 8.0), 0.1 % Tween^®^ 20, 10 % dextran sulfate] for 1 h at 37°C on a polylysine coated slide. The Cy3-labeled snRNA probe was diluted to a final concentration of 5 mM/μl in the Hybmix buffer and hybridization was performed overnight (12–14 h) at 37°C. One well for each sample only contained the hybridization solution, without the Cy3-labeled probe, and was used as a background control. After hybridization, cells were washed with 2× SSC (0.3 mM NaCl, 30 mM sodium citrate, pH 7.0) and 1× SSC, each for 1 h at room temperature, and 0.5× SSC at 37°C and room temperature, each for 30 min, respectively. DNA was stained with 4′, 6-diamidino-2-phenylindole (DAPI) (1:10 000 diluted in 1× PBS) (Merck) for 5 min. The slides were immediately washed once with 1× PBS containing 0.1% Tween-20 and twice with 1× PBS at room temperatures each time for 5 min. Microscopy studies were analyzed under a DFC360 FX camera and the LAS AF 2.7.3.9 software (Leica). Quantification of signals was done with the FIJI-software of 20–30 cells per analysis. Generally, the signal of the nucleus was subtracted from that of the whole cell.

### Cytosolic fractionation and RNA co-IP (CRIP)

800 ml of yeast cells were grown to mid log phase at 25°C. The pellet was washed once with 2 ml of YPD medium containing 1 M sorbitol and 2 mM DTT, and afterward resuspended with 2 ml YPD medium, containing 1 M sorbitol and 1 mM DTT. The cell suspension was digested with 10 μl Zymolase (100 mg/ml) at room temperature until at least 50% of the cells appeared gray under the light microscope. The cells were washed once and then incubated at 25°C for 30 min in 50 ml of YPD medium containing 1 M sorbitol without DTT. Afterward, the cells were shifted to 37°C for 1 h. 1 ml of the total lysate was used for SDS–PAGE and western blot, while 49 ml of the culture were used for the cytosolic fraction experiment. For the total fraction, the cells were lysed with PBSKMT buffer utilizing a FastPrep machine (MP Biomedicals). For the cytosolic fraction, cells were gently resuspended in 1 ml buffer A (50 mM NaCl, 1 mM MgCl_2_, and 10 mM HEPES, pH 6.0) and 0.5 ml of lysis buffer (18% Ficoll 400, 10 mM HEPES, 1 µl of Ribolock, 1 µl of PMSF, and 1 µl of 50× protein inhibitor stock, pH 6.0). After vortexing, the mixtures were centrifuged twice at 12 500 rpm 4°C for 10 min until it the supernatant was clear. The supernatant was collected as the cytosolic fraction. 50 µl of cytosolic fraction was taken for SDS–PAGE and western blot analysis and 100 µl of the cytosolic fraction was used to serve as input samples for the RNA isolation. Nop1 protein was detected as nuclear marker and the Zwf1 served as cytosolic marker by using individual antibodies in western blot analysis. Subsequently, the remaining cytosolic fraction for the RIP experiments was incubated with 20 µl of GFP-trap beads in 10 ml RIP buffer containing 0.02 U per ml RiboLock and 1 μl of blue glycogen (Thermo Fisher Scientific) overnight at 4°C with agitation. Finally, RIP experiments, cDNA synthesis and qPCR detection were performed as described for the RIP experiment.

### Site-directed mutagenesis

A PCR-based mutagenesis was employed to introduce the point mutation and partial deletion in U6 as described previously [54]. The gene under its endogenous promoter with 1 kb upstream and downstream flanking regions was amplified from genomic DNA of a wild-type strain (HKY36). The sequence was cloned into a plasmid with a *URA3* marker (pHK1725). Forward and reverse primer pairs containing the point mutation or the deletion were used for amplification. To degrade the parental plasmids after the PCR, *DpnI* was added with 0.4 U/µl and incubated for 10 h at 37°C. The PCR product was dialyzed and used for transformation of yeast cells in wild-type. Subsequently, genomic DNA was extracted from the positive clones and sequenced to confirm the generated mutants (pHK1942 and pHK1943).

### GFP-microscopy

The procedure was carried out as described earlier [14]. 10 ml of yeast cells were grown to the log phase at 25°C, before shifting them to the nonpermissive temperature. Cells were fixed with 700 μl of 37% deionized formaldehyde and collected through centrifugation at 4000 rpm for 5 min. The cell pellet was washed once with 1 ml of 0.1 M phosphate buffer and 1 ml of P solution, respectively. DNA-staining and microscopy were performed as described in the FISH experiment.

### Domain analysis and homology studies

The amino acid sequences of the indicated proteins were downloaded from the UniProt database (https://www.uniprot.org/). An online tool was used to analyze the main domain of each protein from the SMART database (http://smart.embl-heidelberg.de/). http://smart.embl-heidelberg.de/). The similarities of the amino acid sequences of proteins were calculated by the software Clustal 2.1. The online ClustalW tool (https://www.genome.jp/tools-bin/clustalw) was used to align multiple protein sequences, and the alignment result was then visualised using the ESPript 3.0 tool (https://espript.ibcp.fr/ESPript/ESPript/index.php).

### Recombinant Xrn1 digestion

Total RNA was isolated from wild-type and the *cet1-2* mutant cells using via TRIzol–phenol–chloroform (Thermo Fisher Scientific) extraction after a temperature shift to 37°C for 2 h. The isolated RNA was incubated with the 0.5 μl of RiboLock RNase Inhibitor (Thermo Scientific) and the recombinant exonuclease Xrn1 (New England Biolabs) for 2 h at 37°C, followed by cDNA synthesis (NIPPON Genetics). Subsequent qPCR analysis was performed with the indicated genes.

### 3′ end PCR

Total RNA was isolated from log phase wild-type and the *mex67-5 xpo1-1* and *cse1-1 mtr10*∆ mutants via TRIzol–phenol–chloroform extraction after a temperature shift to 37°C for 1 h. The 0.5 μl RiboLock RNase Inhibitor (Thermo Scientific) was added to the isolated RNA. 5 μmol ssDNA adapter (HK6040) was ligated to the 3′ end of pre-U6 in addition of T4 RNA Ligase (New England Biolabs) at 37°C for 2 h. The specific primer HK6039 was used to synthesize cDNA (NIPPON Genetics). Subsequent qPCR analysis with a forward and reverse primer was performed with the indicated genes.

### Specific downregulation of U6

The procedure was essentially done as previously reported [48]. Site-directed mutagenesis was used to change the wild-type *TATA* box (the consensus sequence is TATAAA) in the yeast U6 promoter region to *TGTA*. This mutation results in the transcriptional down regulation of U6, which is lethal to the cells. Three amino acid substitutions in the yeast TATA box-binding protein Spt15 restores the DNA-binding specificity in the *U6 TGTA* box mutant. The amino acids at positions 194 (Isoleucine), 203 (Valine), and 205 (Leucine) in Spt15 were replaced with Phenylalanine, Threonine and Valine, respectively, resulting in the mutSpt15 protein through constructing a *mutSPT15* expression vector with a *Tet:off* promoter that drives the *mutSPT15 TGTA box* (resulting in the strain HKY2974). The addition of 100 µg/ml doxycycline to the growth medium downregulated the *mutSPT15* expression, resulting in a decrease in U6 expression.

### Quantification and statistical analysis

All experiments in this study were conducted in at least three independent biological replicates. The standard deviation is indicated by the error bars. The *P*-values were determined using an unpaired, two-tailed, unequal Student’s *t*-test and are indicated as follows: *** (*P* < 0.001), ** (*P* < 0.01), and * (*P* < 0.05). Images were quantified using the ImageJ software.

## Supplementary Material

gkaf1500_Supplemental_File

## Data Availability

All data were stored at the Gesellschaft fuer wissenschaftliche Datenverarbeitung mbH Goettingen (GWDG) and will be shared on reasonable request to the corresponding author.
