# Supplementary material for: Initial spliceosomal U4/U6 di-snRNA formation occurs in the cytoplasm of Saccharomyces cerevisiae and requires a guard protein mediated quality control
Source: Nucleic Acids Res. 2026 Jan 8;54(1):gkaf1500. doi: 10.1093/nar/gkaf1500 (PMC12781873; doi:10.1093/nar/gkaf1500)
Supplement: gkaf1500_Supplemental_File [file gkaf1500_supplemental_file.pdf]

Supplementary information to:

Initial Spliceosomal U4/U6 di-snRNA formation occurs in the cytoplasm of *S. cerevisiae* and requires a guard protein mediated quality control

Xiaoxiao Wang, Jian Guo, Jing Li and Heike Krebber*

Abteilung für Molekulare Genetik, Institut für Mikrobiologie und Genetik, Göttinger Zentrum für Molekulare Biowissenschaften (GZMB), Georg-August Universität Göttingen, Göttingen, Germany

*Correspondence: heike.krebber@biologie.uni-goettingen.de

Keywords: snRNA maturation / snRNA processing / U6 / RNAP III / Prp24 / guard protein

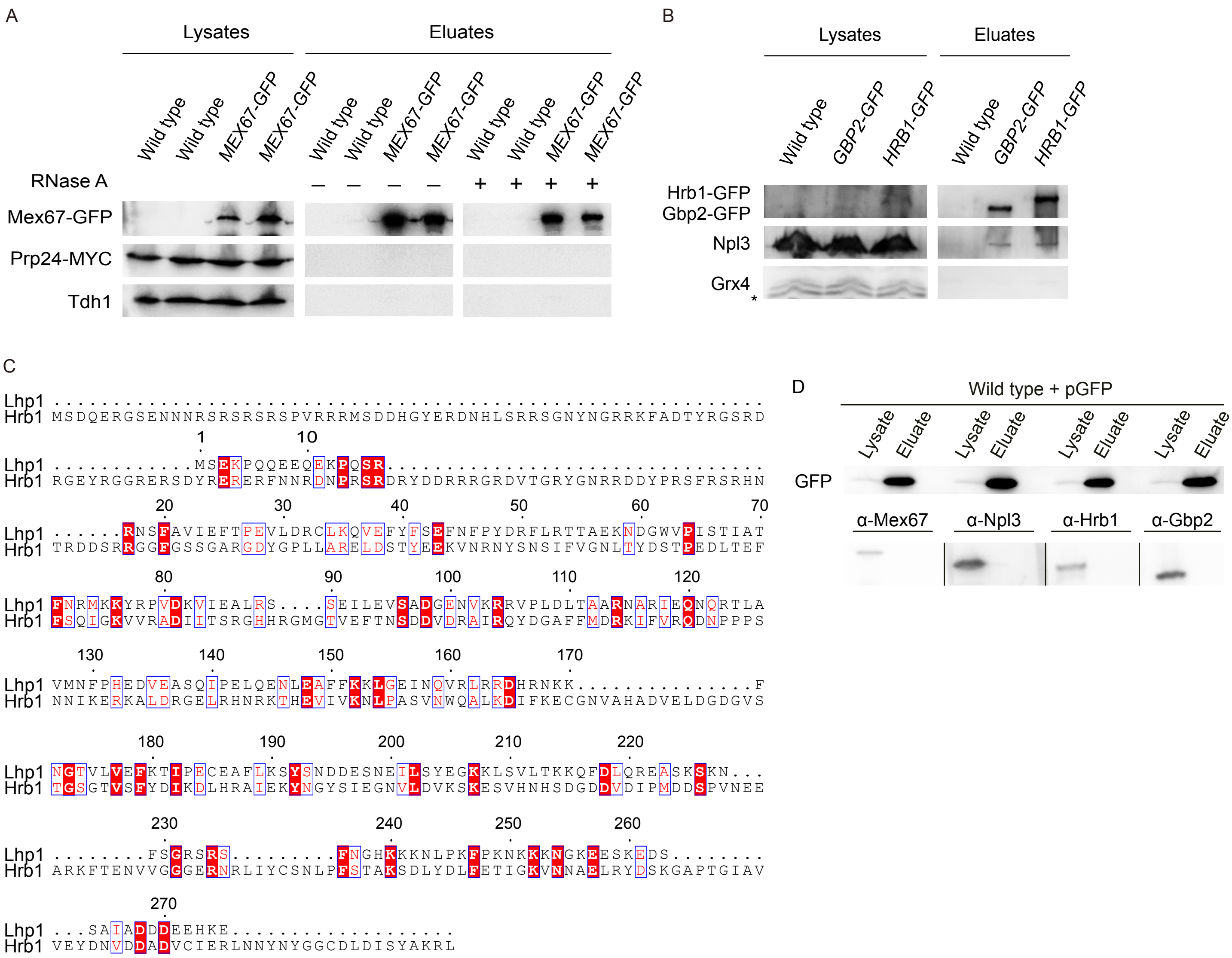

Supplementary Figure 1: Interaction studies with U6 interacting proteins. Related to Figure 1. (A) The mRNA export receptor Mex67 does not interact with Prp24. Western blots of co-immunoprecipitation (IP) analysis of Prp24-myc with Mex67-GFP is shown. The mitochondrial protein Tdh1 served as a washing control for unspecific binding. RNase A was added to one half of the lysates to eliminate the RNA. (B) Western blot of co-IPs of Npl3 with GFP-tagged Hrb1 and Gbp2 are shown. Grx4 served as a washing control for unspecific binding. * indicates an unspecific protein band. (C) Alignment of the amino acid sequences of Lhp1 and Hrb1, indicating identical (red) and conserved residues, related to Figure 1D. (D) Negative control western blot analysis for GFP binding, related to Figure 1A, E-G and Supplementary Figure 1A. IP of GFP expressed in wildtype cells was analyzed for binding to Mex67, Npl3, Hrb1 and Gbp2.

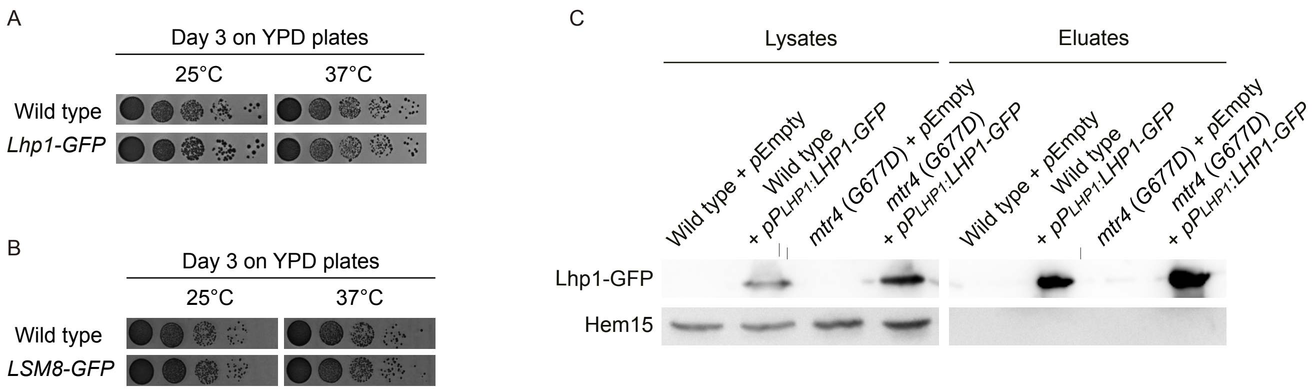

Supplementary Figure 2: Control experiments, related to Figure 2J. (A) GFP-tagging of Lhp1 does not affect cell growth. Growth was compared to a wild type strain. 10-fold serial dilutions were spotted onto full medium plates. (B) The GFP-tagged Lsm8 strain grows like wild type. 10-fold serial dilutions were spotted onto full medium plates. (C) Western blot analysis of Lhp1 pull down shows equal pull down. The indicated strains were grown to log phase and shifted to 37°C 1h before lysis. Hem15 served as a washing control.

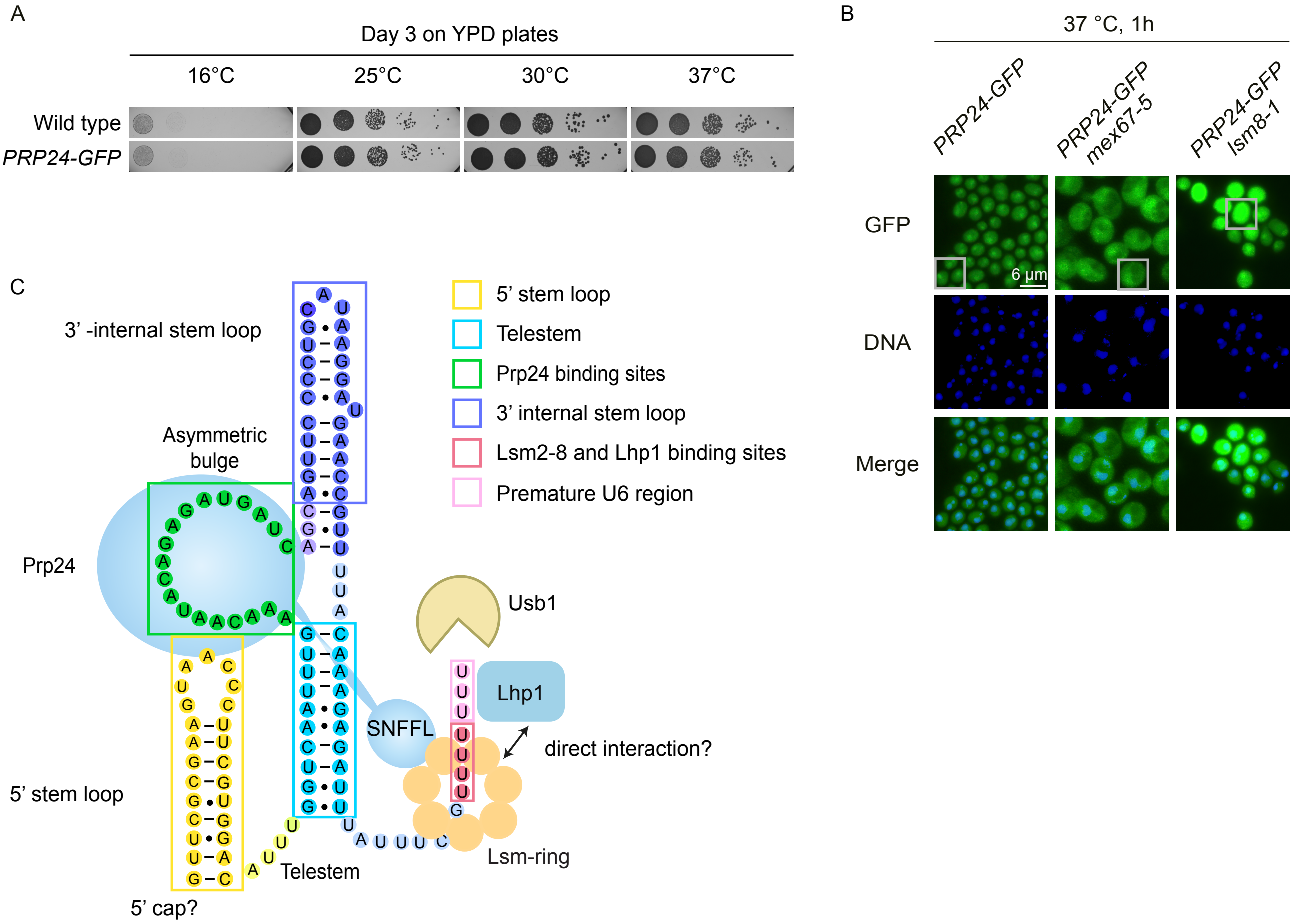

Supplementary Figure 3: Prp24-mediated annealing of pre-U4 and pre-U6 occurs in the cytoplasm. Related to Figure 4. (A) The Prp24-GFP is functional. All yeast strains were spotted in 10-fold serial dilution onto full medium containing agar plates at the indicated temperatures and their growth is documented. (B) Overview over several cells shown in Figure 4A. Prp24-GFP mislocalizes to the cytoplasm in the indicated strains. (C) Overview of U6 and associated proteins.

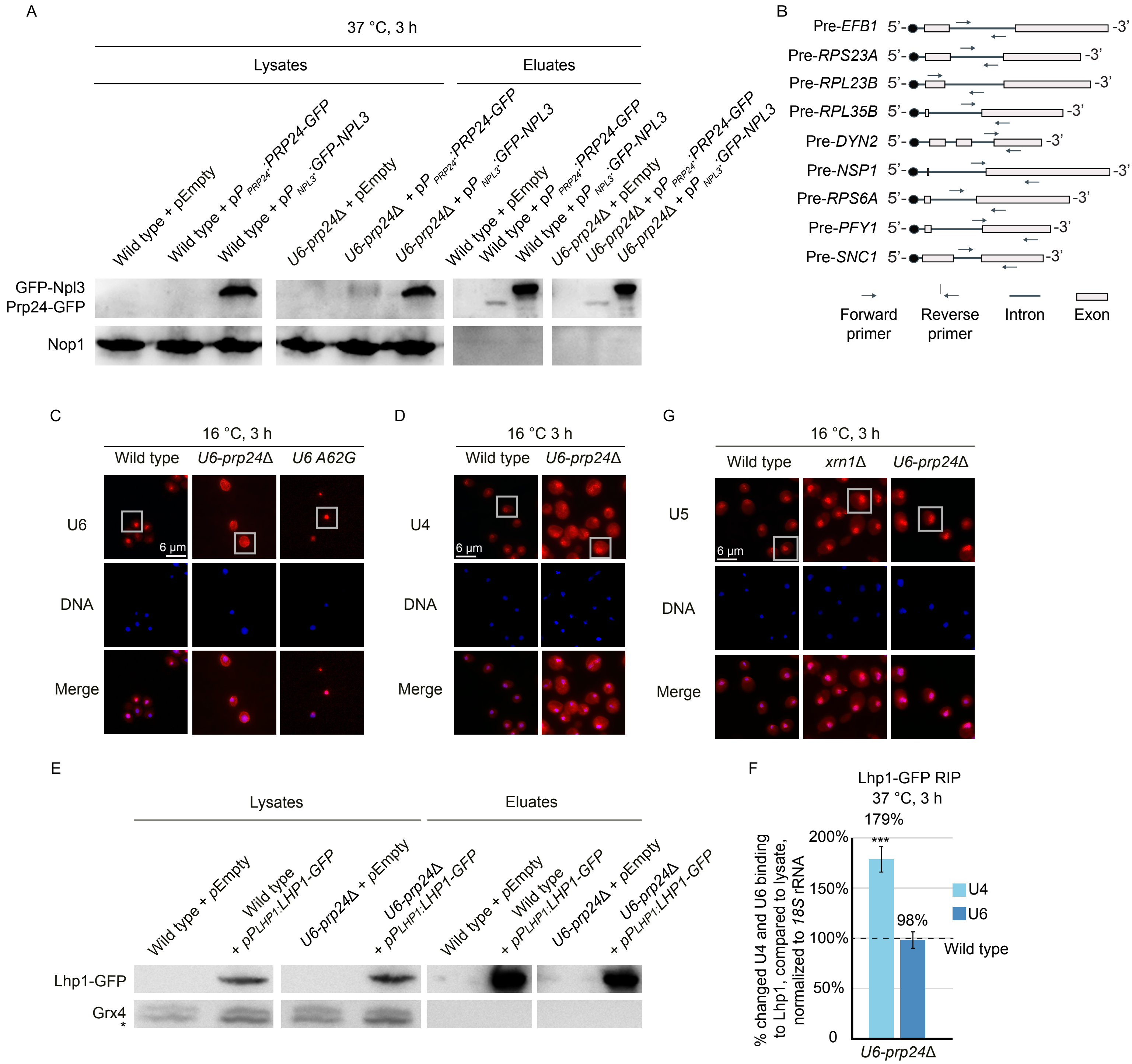

Supplementary Figure 4: Mutation of U6 that prevent di-snRNP formation result in Prp24, U6 and U4 mislocalization. Related to Figure 5. (A) Western blot analysis of the co-IP of GFP-Npl3 and Prp24-GFP is shown in the indicated strains. Nop1 served as a washing control for unspecific binding. (B) Scheme of the primers used to amplify the introns of several randomly chosen example genes. (C/D) Overview over several cells shown in Figure 5E, F, respectively. FISH experiments with the Cy3 labelled specific probes for U4 and U6 are shown in the indicated strains. (E/F) The binding of U6-prp24Δ to Lhp1 is not disturbed. Western blot analysis of an example pulldown is shown in (E), which was used for the RIP experiment shown in (F). (G) Overview over several cells shown in Figure 5H. FISH experiments with the Cy3 labelled specific probes for U5 are shown in the indicated strains.

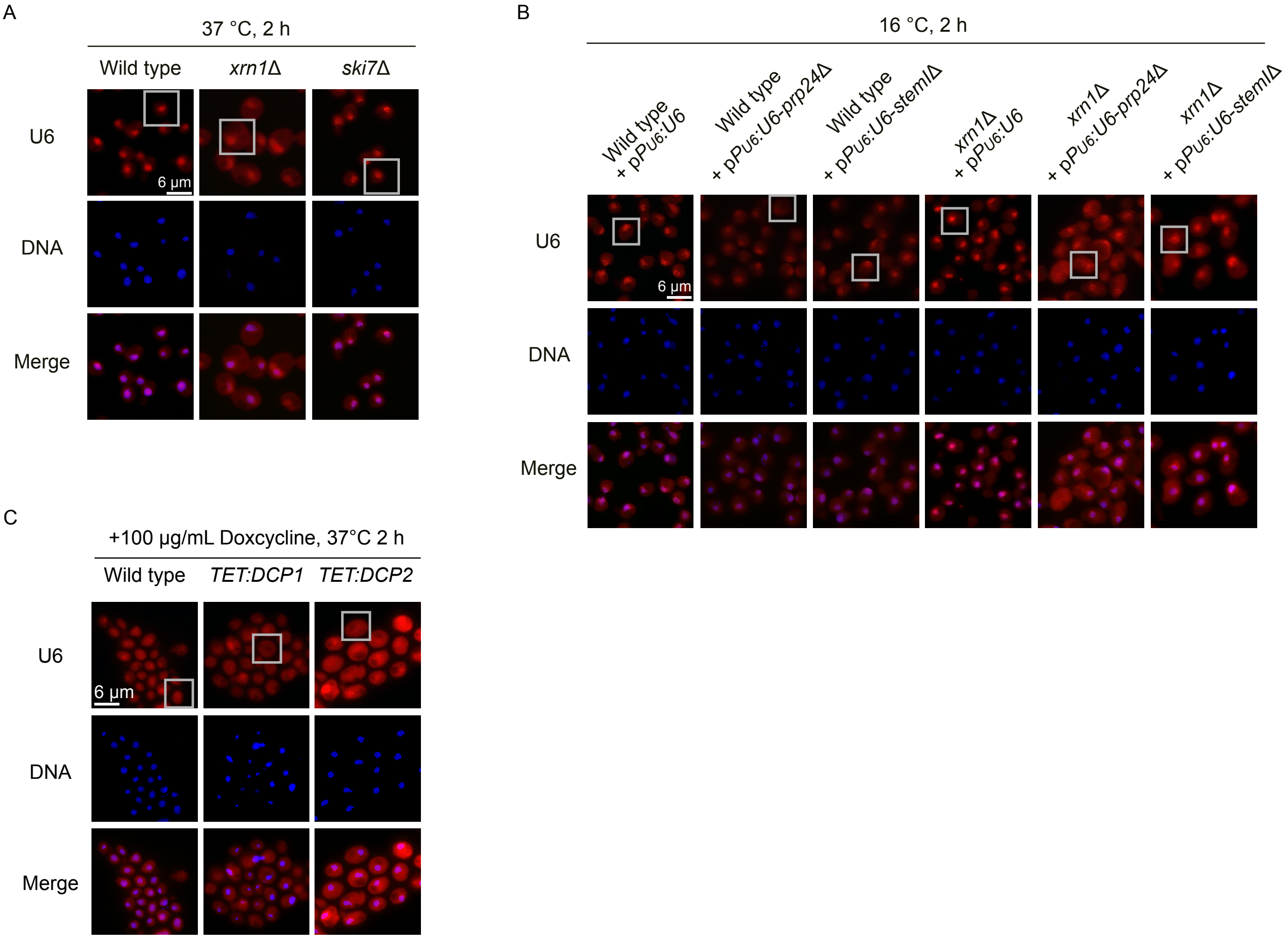

Supplementary Figure 5: Faulty U4/U6-di snRNP formation is detected in the cytoplasm and defective U6 is eliminated from its 5' end. Related to Figure 6. (A) Overview over several cells shown in Figure 6A. U6 accumulates in the cytoplasm of xrn1Δ. FISH experiments with a Cy3 specific U6 probe was carried out in the indicated strains. (B) Overview over several cells shown in Figure 6B. FISH experiments with a Cy3-labelled specific U6 probe were carried out in the indicated strains. (C) Overview over several cells shown in Figure 6G. FISH experiments with a Cy3-specific U6 probe were carried out in the cells containing either a tetracycline down-regulatable DCP1 or DCP2 gene, that were treated with doxycycline at the non-permissive temperature.

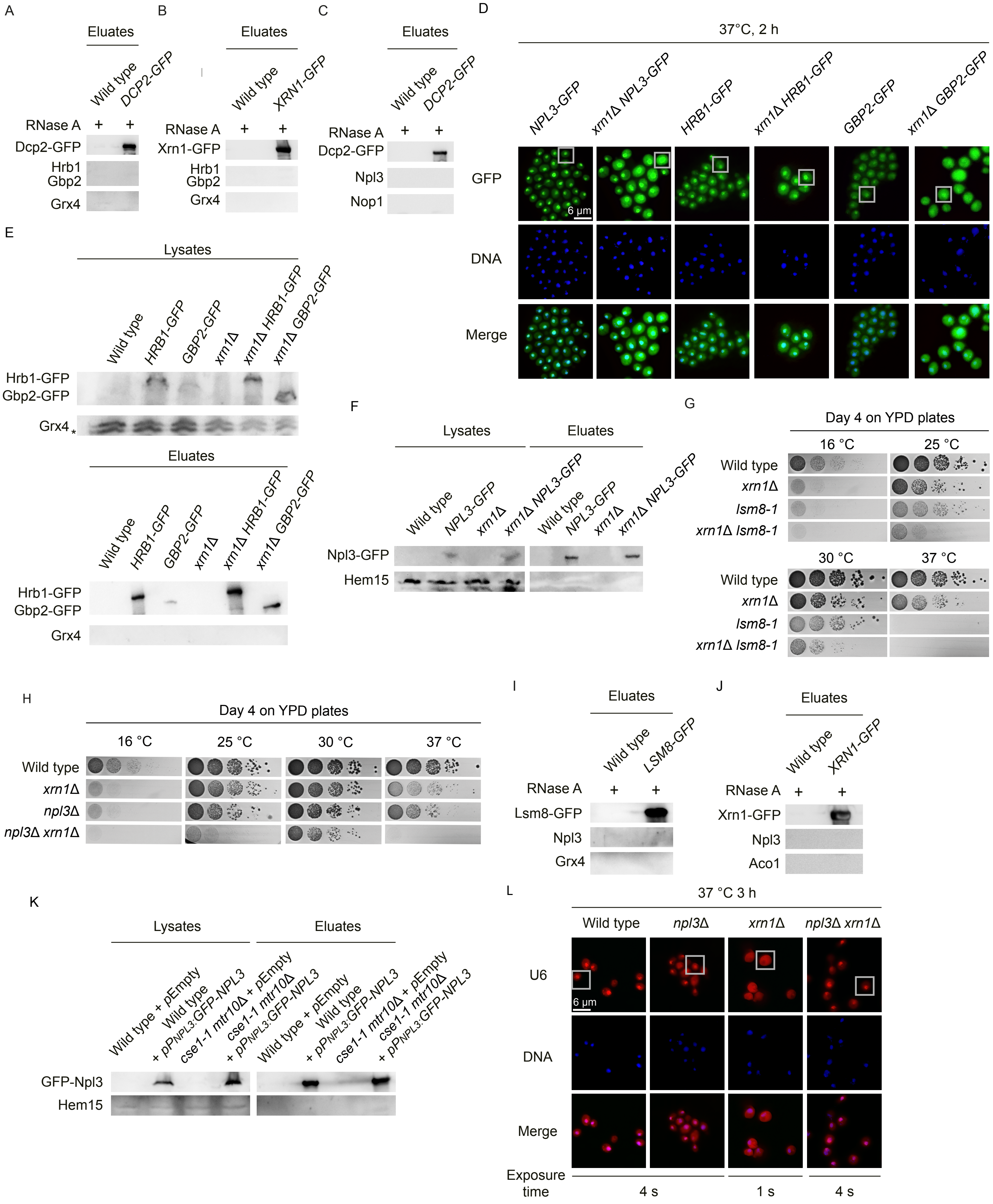

Supplementary Figure 6: Physical and genetic interactions of the guard proteins and the cytoplasmic degradation machinery. Related to Figure 7. (A-C) Western blot analysis of RNase treated samples of the pulldown experiments shown in Figure 7A-C. (D) Overview over several cells shown in Figure 7D. (E, F) Western blot analysis of an example pulldowns of Hrb1- and Gbp2-GFP or GFP-Npl3, respectively in wildtype and xrn1Δ shifted for 1h to 37°C are shown. Grx4 served as negative control and the asterisk indicates an unspecific band for Gbp2 and Hrb1 and Hem15 served as washing control for Npl3. (Related to Figure 7E) (G) Genetic interaction between mutants of LSM8 and XRN1. 10-fold serial dilutions

were spotted onto full medium plates. (H) Genetic interaction between mutants of NPL3 and XRN1. 10-fold serial dilutions were spotted onto full medium plates. (I, J) Npl3 does not interact with Lsn8 (I) or Xrn1 (J). (Related to Figure 7I, J) (K) Western blot analysis of the IP of GFP-Npl3 for the RIP experiment shown in Figure (7K) is shown in the indicated strains. Hem15 served as a washing control for unspecific binding. (L) Overview over several cells shown in Figure (7M).

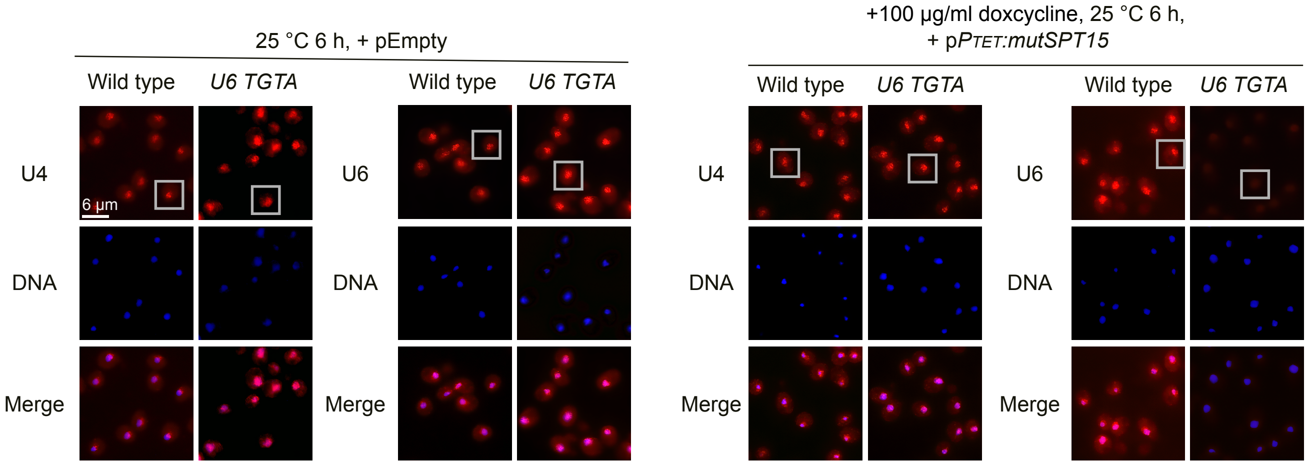

Supplementary Figure 7: Overview of several cells shown in Figure 8B. Cells were treated as described in Figure 8B.

Supplementary Tables

Supplementary Table 1. ***Saccharomyces cerevisiae*** **strains used in this study.**

Number	Genotype	Source
HKY36	*MATα ura3-52 leu2Δ1 his3Δ200*	(Winston et al.,1995)
HKY165	*MATα ura3-52 leu2Δ1 his3Δ200 lys2 hrb1::HIS3*	(Hackmann et al, 2014)
HKY169	*MATα ura3 leu2 his3 lys1 ade2 ade8 gbp2::HIS3 hrb1::HIS3*	(Hackmann et al, 2014)
HKY206	*MATα ade2-1 his leu trp1-1 ura3-1 xpo1::LEU2 + p CEN TRP1 xpo1-1*	(Taura et al., 1998)
HKY208	*MATα ura3-52 ade2-101 his3Δ1 trp1- Δ901 cse1-1*	(Xiao et al., 1993)
HKY257	*MATα ura3 leu2 his3 gbp2::HIS3*	(Hackmann et al, 2014)
HKY270	*MATα ura3 leu2 his3 ade2 mtr10::HIS3 + p CEN TRP1 mtr10-7*	(Senger et al., 1998)
HKY501	*MATa his3Δ1 leu2Δ0 met15Δ0 ura3Δ0 Hrb1-GFP:HIS3MX6*	Invitrogen (Carlsbad/USA)
HKY502	*MATa his3Δ1 leu2Δ0 met15Δ0 ura3Δ0 GBP2-GFP:HIS3MX6*	Invitrogen (Carlsbad/USA)
HKY644	*MATα ura3 leu2 trp1 his3Δ ade2 mex67::HIS3 + p CEN LEU2 mex67-5*	(Segref et al., 1997)
HKY682	*MATα his3Δ1 leu2Δ0 ura3Δ0 LYS TRP ADE npl3::KanMX4*	(Klama et al. 2022)
HKY719	*MATα his3Δ1; leu2Δ0; ura3Δ0; LYS2; TRP; ADE npl3::kanMX4 gbp2::kanMX4 hrb1::kanMX4 + p CEN URA3 NPL3*	(Zander et al. 2016)
HKY1240	*MATα ura3Δ0 leu2Δ0 his3Δ1 xrn1::kanMX4*	Invitrogen (Carlsbad/USA)
HKY1241	*MATα ura3Δ0 leu2Δ0 his3Δ1 met15Δ0 ski7::kanMX4*	Invitrogen (Carlsbad/USA)
HKY1277	*MATa his3Δ1 leu2Δ0 met15Δ0 ura3Δ0 MTR10-GFP::HIS3MX6*	Invitrogen (Carlsbad/USA)
HKY1353	*MATa ura3-52 mex67::HIS3 xpo1::TRP1 xpo1-1::HIS + p CEN LEU2 mex67-5*	(Brune et al., 2005)
HKY1509	*ura3Δ0 leu2Δ0 his3Δ1 met15Δ0 cet1-2*	(Klama et al. 2022)
HKY1596	*MATa his3Δ1 leu2Δ0 met15Δ0 ura3Δ0 CSE1-GFP:HIS3MX6*	Invitrogen (Carlsbad/USA)
HKY1854	*MATa ura3 lys2 ade2 trp1 his3 leu2 lsm8-1*	(Pannone et al. 1998)
HKY1863	*MATa his3Δ1 leu2Δ0 met15Δ0 ura3Δ0 Lhp1-GFP:HIS3MX6*	Invitrogen (Carlsbad/USA)
HKY1880	*MATa his3Δ1 leu2Δ0 met15Δ0 ura3Δ0 DCP1-GFP:HIS3MX6*	Invitrogen (Carlsbad/USA)
HKY1906	*MATa leu2Δ0 his3Δ1 met15Δ0 lhp1::kanMX4*	Invitrogen (Carlsbad/USA)
HKY2109	*MATa his3Δ1 leu2Δ0 met15Δ0 ura3Δ0 LSM8::GFP:HISMX6*	Invitrogen (Carlsbad/USA)
HKY2087	*MATα -his -ura KAN cse1-1 mtr10:kanMX4*	(Hirsch et al. 2021)
HKY2208	*MATa leu2Δ0 his3Δ1 met15Δ0 xrn1::kanMX4*	Invitrogen (Carlsbad/USA)
HKY2736	*MATa his3Δ1 leu2Δ0 DCP1::kanR-tet07-TATA URA3::CMV-Tta*	Hughes TET promoters collection
HKY2737	*MATa his3Δ1 leu2Δ0 DCP2::kanR-tet07-TATA URA3::CMV-Tta*	Hughes TET promoters collection
HKY2738	*MATa his3Δ1 leu2Δ0 met15Δ0 ura3Δ0 DCP2::GFP:HISMX6*	Invitrogen (Carlsbad/USA)
HKY2742	*MATα leu2Δ0 ura3Δ0 PRP24-GFP:HIS3MX6*	This study
HKY2759	*MATa ura3Δ0 npl3::KanMX4 XRN1-GFP:HIS3MX6*	This study
HKY2761	*MATa leu2Δ0 xrn1::KanMX4 NPL3-GFP:HIS3MX6*	This study
HKY2762	*MATa leu2Δ0 xrn1::KanMX4 HRB1-GFP:HIS3MX6*	This study
HKY2763	*MATa ura3Δ0 leu2Δ0 xrn1::KanMX4 GBP2-GFP:HIS3MX6*	This study
HKY2764	*MATa his3Δ1 lys2-301 trp1 leu2Δ0 ura3Δ0 xrn1::KanMX4 npl3::KanMX4*	This study

HKY2766	*MATa his3Δ1 trp1 leu2Δ0 ura3Δ0 lsm8-1 npl3::KanMX4*	This study
HKY2767	*MATa ura3Δ0 mex67::HIS3 LSM8-GFP:HIS3MX6 (+pUN100-mex67-5-LEU)*	This study
HKY2768	*MATα ura3Δ0 leu2Δ0 lsm8-1 PRP24-GFP:HIS3MX6*	This study
HKY2769	*MATα leu2Δ0 U6-prp24Δ*	This study
HKY2770	*MATα leu2Δ0 U6- prp24Δ*	This study
HKY2771	*MATα leu2Δ0 U6 A62G*	This study
HKY2774	*MATa his3Δ1 leu2Δ0 ura3Δ0 ade2 lhp1::KanMX4 npl3::KanMX4*	This study
HKY2777	*MATα lys2 ade2 lhp1::KanMX4 hrb1::HIS*	This study
HKY2778	*MATa his3Δ1 leu2Δ0 lys2 ade2 lhp1::KanMX4 gbp2::HIS*	This study
HKY2780	*MATα ura3Δ0 leu2Δ0 lsm8-1 USB1-GFP:HIS3MX6*	This study
HKY2791	*MATa ura3Δ0 PRP24-GFP:HIS3MX6 mex67::HIS +p CEN LEU mex67-5*	This study
HKY2792	*MATα ura3Δ0 PRP24-GFP:HIS3MX6 mex67::HIS +p CEN LEU mex67-5*	This study
HKY2783	*MATα ade2 cse1-1 mtr10::KanMX4 +p CEN URA mtr10-7*	This study
HKY2802	*MATα ade2 mex67::HIS3 xpo1::LEU2 +p CEN LEU mex67-5, +p CEN TRP1 xpo1-1*	This study
HKY2806	*MATα trp1 cse1-1 PRP24-GFP:HIS3MX6*	This study
HKY2974	*MATα ura3-52 leu2Δ1 his3Δ200 U6 TGTA Δ+p CEN LEU* $P_{Tet\text{-}off:}$ *mutSPT15*	This study

Supplementary Table 2. Plasmids used in this study.

Number	Genotype	Source
pHK87	*LEU2, CEN, AMP*^{R}	(Sikorski et al., 1989)
pHK88	*URA3, CEN, AMP*^{R}	(Sikorski et al., 1989)
pHK453	$P_{MTR10:}$*mtr10-7, CEN, TRP, AMP*^{R}	(Senger et al., 1998)
pHK1705	$P_{ADH1:}$*Lsm8-GFP, CEN, URA3, AMP*^{R}	(Gianluca Zaccagnini)
pHK1708	$P_{ADH1:}$*Lsm8-MYC, CEN, URA3, AMP*^{R}	(Gianluca Zaccagnini)
pHK1711	$P_{Lsm8:}$*Lsm8-GFP, CEN, URA3 ,AMP*^{R}	(Gianluca Zaccagnini)
pHK1712	$P_{Lsm8:}$*Lsm8-MYC, CEN, URA3, AMP*^{R}	(Gianluca Zaccagnini)
pHK1746	$P_{ADH1:}$*LHP1-GFP, CEN, URA3, AMP*^{R}	(Gianluca Zaccagnini)
pHK1747	$P_{ADH1:}$*LHP1-MYC, CEN, URA3, AMP*^{R}	(Gianluca Zaccagnini)
pHK1748	$P_{LHP1:}$*LHP1-GFP, CEN, URA3, AMP*^{R}	(Gianluca Zaccagnini)
pHK1749	$P_{LHP1:}$*LHP1-MYC, CEN, URA3, AMP*^{R}	(Gianluca Zaccagnini)
pHK1994	$P_{MTR10:}$*mtr10-7, CEN, URA, AMP*^{R}	This study
pHK2001	$P_{PRP24:}$*PRP24-3xMYC, CEN, URA3, AMP*^{R}	This study
pHK2002	$P_{PRP24:}$*PRP24-GFP, CEN, URA3, AMP*^{R}	This study
pHK2003	$P_{ADH1:}$*PRP24-3xMYC, CEN, URA3, AMP*^{R}	This study
pHK2004	$P_{ADH1:}$*PRP24-GFP, CEN, URA3, AMP*^{R}	This study
pHK1725	*URA3, CEN, AMPR, LOXP AMP*^{R}	This study
pHK1941	$P_{U6:}$*U6, URA3, CEN, LOXP AMP*^{R}	This study
pHK1942	$P_{U6:}$*U6-prp24Δ, URA3, CEN, LOXP AMP*^{R}	This study
pHK1943	$P_{U6:}$*U6-stemIΔ, URA3, CEN, LOXP AMP*^{R}	This study
pHK1945	$P_{U6:}$*U6 A62G, URA3, CEN, LOXP AMP*^{R}	This study
pHK2047	$P_{U6:}$*U6, URA3, CEN, AMP*^{R}	This study
pHK2048	$P_{U6:}$*U6-stemIΔ, URA3, CEN, AMP*^{R}	This study
pHK2049	$P_{U6:}$*U6 A62G, URA3, CEN, AMP*^{R}	This study
pHK2198	$P_{ADH1:}$*GFP, URA3, CEN, AMP*^{R}	This study
pHK2210	$P_{Tet\text{-}off:}$ *mutSPT15, LEU, CEN AMP*^{R}, Tet-off motifs: *CMV-rTA-ADH1 terminator*	This study

Supplementary Table 3. Oligonucleotides used in this study.

Number	Sequence (5' to 3')	Target
HK778	5'-GGTCCGTGTTTATGATGG-3'	*EFB1* intron forward
HK779	5'-CTCTGCTCTGTGCTATCG-3'	*EFB1* intron reverse
HK874	5'-TCACCAACGGCTTCACCATC-3'	*RPS6A* intron reverse
HK1093	5'-GGTTTACATATACTGTTACTACAAACC-3'	*RPS6A* intron forward
HK1396	5'-CATGGCCGTTCTTAGTTGGTGG-3'	*18S* rRNA forward
HK1397	5'-ATTGCCTCAAACTTCCATCGGC-3'	*18S* rRNA reverse
HK1404	5'-TCGCGAAGTAACCCTTCGTG-3'	*U6*/*SNR6* forward
HK1405	5'-AAACGGTTCATCCTTATGCAGG-3'	*U6*/*SNR6* reverse
HK1608	5'-TATCAGAGGAGATCAAGAAGTCCTA-3'	*U1*/*SNR19* forward
HK1628	5'-CTATCAACCCCCTATTGTGATATCG-3'	*RPS23A* intron forward
HK1629	5'-GATTGGGAAGAATGGCCGAAGC-3'	*RPS23A* intron reverse
HK1707	5'-TGAGGTCCCGCATGAATGAC-3'	*21S* rRNA forward
HK1708	5'-GGGTCTTTCCGTCTTGCTGA-3'	*21S* rRNA reverse
HK1709	5'-ACGGTTATAAGAACGTTCAACGAC-3'	*21S* rRNA intron forward
HK1710	5'-GGGCGCTCGTGGATGAATTA-3'	*21S* rRNA intron reverse
HK1738	5'-TGCAAACTCCTTGGTCACAC-3'	*U1*/*SNR19* forward
HK1739	5'-CCAGGCAGAAGAAACAAAGG-3'	*U1*/*SNR19* reverse
HK1740	5'-AGCCATGACTGCATCTGTTG-3'	*U2*/*LSR1* forward
HK1741	5'-ACAGGCGTCAACCATCAAG-3'	*U2*/*LSR1* reverse
HK1752	5'-CCTTATGCACGGGAAATACG-3'	*U4*/*SNR14* forward
HK1765	5'-Cy3-CGGTTCATCCTTATGCAGGGGAACTGCTGA TCATCTCTGTATTGTTTCAA-Cy3-3'	*U6*/*SNR6* probe
HK2154	5'-CCAGAACAATCCGTACACAAGG-3'	*HEM15* forward
HK2155	5'-GCAATTGTCTTCTGATACTTAGCAC-3'	*HEM15* reverse
HK2857	5'-GTATTCAAAAGCGAACACCG-3'	*U4*/*SNR14* reverse
HK2859	5'-CAGCTTTACAGATCAATGGC-3'	*U5*-L/*SNR7-L* forward
HK2860	5'-TATGGCAAGCCCACAGTAA-3'	*U5-L*/*SNR7-L* reverse
HK2867	5'-CGGACAGCTTTACCTGTTTCTATGGAGACAACACCCGGAT GGTTCTGGTA-Cy3-3'	*U5-L*/*SNR7-L* probe
HK2957	5'-CCTGCGAACCTGTAAACTAC-3'	*NSP1* intron forward
HK2956	5'-GTTATTGCTACCAAATGCAGG-3'	*NSP1* reverse
HK2969	5'-CTAACAAGGAGAAAACTTAGACC-3'	*DYN2* intron forward
HK2968	5-'CAATCACATGCCAGGTATTGC-3'	*DYN2* reverse
HK2972	5'-CAGCATACACTGATAACTTAATAG-3'	*PFY1* intron forward
HK2971	5'-CAGCATCATGTCTACCGTAG-3'	*PFY1* reverse
HK2975	5'-GCATTGCACAGAGCGTATTAG-3'	*RPL35B* intron forward
HK2974	5-'GGTCTGGACAACTTTTGGAC-3'	*RPL35B* reverse
HK2981	5'-CATTCATATACGTTCTTCGTG-3'	*SNC1* intron forward
HK2980	5'-CTAAAGCCAGACACATCTTC-3'	*SNC1* reverse
HK2985	5'-AGGAGACGGTCTGGTTTATAATTAAATTTCAACCAGCAA AAAC-Cy3-3'	*U4*/*SNR14* probe
HK5114	5'-GGTCAATTTGAGAGATGATCAGCAGTTCCCCTGCA-3'	*U6-prp24*Δ forward
HK5115	5'- TGATCATCTCTCAAATTGACCAAATGTCCACGAAGG-3'	*U6-prp24*Δ reverse
HK5187	5'-ATGATCAGCGGTTCCCCTGCATAAG-3'	*U6 A62G* forward
HK5188	5'-AGGGGAACCGCTGATCATCTCTGTATTG-3'	*U6 A62G* reverse
HK5263	5'-GTCAGGTAACGGTGCTCAAGG-3'	*RPL23B* intron forward
HK5264	5'-AAGGCCCAGAAGTAGATTTCGCTATC-3'	*RPL23B* intron reverse
HK6039	5'-GCATACATTATACGAAGTTATGGATCCAAAAAA-3'	Unprocessed *U6*
HK6040	5'-GGATCCATAACTTCGTATAATGTATGCTATACGAAGTTAT -3'	Adaptor for 3'end PCR

Supplementary References

1. Winston F., Dollard C., Ricupero-Hovasse SL. Construction of a set of convenient *Saccharomyces cerevisiae* strains that are isogenic to S288C. *Yeast* (*Chichester, England*). 11, 53-55 (1995).
2. Hackmann A., Wu H., Schneider U.-M., Meyer K., Jung K., and Krebber H. Quality control of spliced mRNAs requires the shuttling SR proteins Gbp2 and Hrb1. *Nature Communications*. 5, 3123 (2014).
3. Taura T., Krebber H., Silver PA. A member of the Ran-binding protein family, Yrb2p, is involved in nuclear protein export. *Proceedings of the National Academy of Sciences of the United States of America*. 95, 7427-7432 (1998).
4. Xiao Z., McGrew JT., Schroeder AJ., Fitzgerald-Hayes M. CSE1 and CSE2, two new genes required for accurate mitotic chromosome segregation in *Saccharomyces cerevisiae*. *Molecular and Cellular Biology*. 13, 4691-4702 (1993).
5. Senger B., Simos G, Bischoff FR., Podtelejnikov A., Mann M., Hurt E. Mtr10p functions as a nuclear import receptor for the mRNA-binding protein Npl3p. *The EMBO Journal*. 17(8), 2196-207 (1998).
6. Segref A., Sharma K., Doye V., Hellwig A., Huber J., Lührmann R., Hurt E. Mex67p, a novel factor for nuclear mRNA export, binds to both poly(A)+ RNA and nuclear pores. *The EMBO journal* 16, 3256-3271 (1997).
7. Zander G., Hackmann A., Bender L., Becker D., Lingner T., Salinas G., and Krebber H. (2016). mRNA quality control is bypassed for immediate export of stress-responsive transcripts. *Nature*. 540, 593-596.
8. Brune C., Munchel SE., Fischer N., Podtelejnikov AV., Weis K. Yeast poly(A)-binding protein Pab1 shuttles between the nucleus and the cytoplasm and functions in mRNA export. *RNA*. 11(4), 517-531 (2005).
9. Klama S., Hirsch A.G., Schneider U.M., Zander G., Seel A., Krebber H. A guard protein mediated quality control mechanism monitors 5'-capping of pre-mRNAs. *Nucleic Acids Research*. 50, 11301-11314 (2022).
10. Pannone B.K., Xue D., Wolin S.L. A role for the yeast La protein in U6 snRNP assembly: evidence that the La protein is a molecular chaperone for RNA polymerase III transcripts. *The EMBO journal*. 17, 7442-7453 (1998).
11. Hirsch A.G., Becker D., Lamping JP., Krebber, H. Unraveling the stepwise maturation of the yeast telomerase including a cse1 and mtr10 mediated quality control checkpoint. *Scientific Reports*. *11*, 22174 (2021).
12. Sikorski RS, Hieter P. A system of shuttle vectors and yeast host strains designed for efficient manipulation of DNA in Saccharomyces cerevisiae. *Genetics* 122, 19-27 (1989).
